# Locomotor Behaviour and Clock Neurons Organisation in the Agricultural Pest *Drosophila suzukii*

**DOI:** 10.3389/fphys.2019.00941

**Published:** 2019-07-24

**Authors:** Celia Napier Hansen, Özge Özkaya, Helen Roe, Charalambos P. Kyriacou, Lara Giongo, Ezio Rosato

**Affiliations:** ^1^Department of Genetics and Genome Biology, University of Leicester, Leicester, United Kingdom; ^2^Centro Ricerca e Innovazione, Fondazione Edmund Mach, Trento, Italy

**Keywords:** circadian clock, *Drosophila*, *suzukii*, SWD, *melanogaster*, circadian rhythms, clock neurons, behaviour

## Abstract

*Drosophila suzukii* (Matsumara) also called Spotted Wing *Drosophila* (SWD), is an invasive pest species originally from Asia that has now spread widely across Europe and North America. The majority of drosophilids including the best known *Drosophila melanogaster* only breed on decaying fruits. On the contrary, the presence of a strong serrated ovipositor and behavioural and metabolic adaptations allow *D. suzukii* to lay eggs inside healthy, ripening fruits that are still on the plant. Here we present an analysis of the rhythmic locomotor activity behaviour of *D. suzukii* under several laboratory settings. Moreover, we identify the canonical clock neurons in this species by reporting the expression pattern of the major clock proteins in the brain. Interestingly, a fundamentally similar organisation of the clock neurons network between *D. melanogaster* and *D. suzukii* does not correspond to similar characteristics in rhythmic locomotor activity behaviour.

## Introduction

In the late 2000s the Asian drosophilid, *Drosophila suzukii* (Matsumura) invaded Europe and North America becoming established in just a handful of years (Asplen et al., 2015). While it is likely that the increasing world trade of goods from Asia was pivotal to the astonishing speed of the invasion, the ability to become widespread in the newly colonised territories speaks volumes about the “plasticity” of this species.

*D. suzukii* is native of Asia and occupies the majority of the continent, from Japan to Russia and from Korea to Pakistan (Calabria et al., 2012; Cini et al., 2012). Although the exact origins are not known, comparative genomics suggests that *D. suzukii* evolved in a region spanning across North India, Indochina, and the Chinese coasts, at a time (9 to 6 Mya) when a mountainous temperate forest was the dominant habitat in this is area (Ometto et al., 2013). *D. suzukii* is part of a species complex within the melanogaster group, hence it is phylogenetically related to the more common and best-known model species *Drosophila melanogaster* (Chiu et al., 2013). However, contrary to *D. melanogaster* and the majority of other drosophilids that only breed and feed on decaying fruits, the presence of a strong serrated ovipositor and novel behavioural and metabolic characteristics (Nguyen et al., 2016; Karageorgi et al., 2017), allow *D. suzukii* to lay eggs inside healthy, ripening fruits that are still on the plant. Damage is firstly caused by the “sting” that leaves a scar on the fruit. Then larvae begin feeding inside the fruit causing the surrounding area to collapse. Thereafter, secondary fungal or bacterial infections contribute to further fruit deterioration (i.e., rotting). These flies have a wide range of hosts although they prefer small fruits such as cherry, strawberry, raspberry, and similar and are able to cause substantial economic losses, in particular to specialised cultivations usually important for local rural economies (Grassi et al., 2009; Goodhue et al., 2011; Cini et al., 2012). In addition, their population explosion aided by the lack of natural enemies in America and Europe constitutes an ecological threat to those regions. Wild fruits, a crucial part of any ecosystem, are under massive attack by this species; directly because of oviposition and larval feeding, and indirectly as a potential vector of plant diseases (Asplen et al., 2015). Curiously, these flies are not considered an important pest in Asia, probably due to the presence of natural predators that preclude the establishment of large populations (Asplen et al., 2015).

The spread of *D. suzukii* has generated widespread interest for its economic impact but also academic attention as a model pest species. Although phylogenetically quite close to *D. melanogaster, D. suzukii* has diverged profoundly due to its revolutionary ecological repositioning as a pest of soft fruits. As mentioned above, this evolutionary change was led by the development of a serrated ovipositor, but behavioural and metabolic plasticity might have provided the premise for such a morphological change to become an ecological innovation (Karageorgi et al., 2017).

To date the best documented changes are in the olfactory system. For instance, unlike other drosphilids, the smell of ripe fruits has become a strong oviposition signal for *D. suzukii* females (Karageorgi et al., 2017). Conversely, they ignore CO_2_, produced during ripening, that is instead a strong repellent for *D. melanogaster* (Krause Pham and Ray, 2015). These differences are also reflected by the evolution and the regulation of olfactory genes (Hickner et al., 2016; Ramasamy et al., 2016; Crava et al., 2019). Further adaptations involve sexual and social behaviour. D *suzukii* does not produce cis-vaccenyl acetate (cVA), a male pheromone (Dekker et al., 2015). The cVA pheromone is used throughout the melanogaster group as an attractant towards females and as a complex social signal towards other males; for the latter, being a repellent or attractant depends on concentration (reviewed in Ejima, 2015). The complexity of cVA signalling is explained by two antagonist olfactory circuits with opposite behavioural valence being activated according to concentration (reviewed in Ziegler et al., 2013). *D. suzukii* flies still perceive cVA but have switched the dominance of the two circuits resulting in a constitutive behavioural repulsion (Dekker et al., 2015). What is the relevance of such a change? Many drosophilids aggregate, mate and oviposit on fermenting fruits and cVA is key to these behaviours (Laturney and Billeter, 2014). Furthermore, fermentation odours, feeding, and cVA are synergistic, acting as a potent aphrodisiac mix (Grosjean et al., 2011; Lebreton et al., 2012; Das et al., 2017). Considering that adult *D. suzukii* flies still use rotting fruits as a food source but exploit fresh fruits as preferential and “niche” mating and oviposition sites (Cloonan et al., 2018), obliterating the production of cVA (not to attract competitor species) and reverting the behavioural response to it (not to be attracted and aroused on the wrong substrate by other species) seems an obligate evolutionary path to be able to succeed in a new larval phytophagous life-style.

More generally, *D. suzukii* and *D. melanogaster* rely on a different sensory representation of the world. *D. suzukii* invests more in the visual system (larger eyes and larger optic lobes in the brain, relative to size) whereas *D. melanogaster* invests more in the olfactory system (more trichoid sensilla—they house sensory neurons detecting pheromones—in the antennae and larger antennal lobes in the brain, relative to size) (Keesey et al., 2019). Such a difference is not a consistent finding in the comparison between *D. suzukii* and the majority of other drosophilids (Keesey et al., 2019); therefore, it is not a consequence of their innovative reproductive strategy *per se*. However, it is symptomatic of the evolutionary feedback between ecological positioning and sensory modalities, which becomes more apparent when comparing species. It affects neuronal numbers and connections providing, for instance, an anatomical basis for the expected divergence in the behavioural repertoires of *D. melanogaster* and *D. suzukii* in line with their different life history strategies. Therefore, as we are interested in circadian behaviour, its evolution and its neuronal basis, we consider comparison of the well-known rhythmic behavioural characteristics of *D. melanogaster* with those of *D. suzukii* to be informative.

The circadian clock is a major player in regulating adaptation to the environment. Patterns of activity/rest, feeding, mating, oviposition and eclosion all show 24 h rhythmicity. This is explained by the existence of a self-sustained timekeeping system that aligns the physiology and the behaviour of the organism with the day/night cycle, irrespective (when within the range tolerated by the species) of random environmental fluctuations in temperature (reviewed in Özkaya and Rosato, 2012). *D. melanogaster* has been pivotal in elucidating the architecture and the logic of animal circadian clocks (Konopka and Benzer, 1971; Bargiello et al., 1984; Zehring et al., 1984). Molecularly, the core of the clock consists of a system of interlocked transcription/translation feedback loops (TTL). The transcription factors CLOCK (CLK) and CYCLE (CYC) bind as heterodimers on the promoters of the *period* (*per*) and *timeless* (*tim*) genes initiating their transcription (Allada et al., 1998; Rutila et al., 1998). Immediately after translation, their protein products, PER and TIM, become a substrate for several kinases and phosphatases, resulting in a complex series of post-translational events (Price et al., 1998; Martinek et al., 2001; Sathyanarayanan et al., 2004; Fang et al., 2007). This progressive “maturation” controls the ability of PER and TIM to dimerise, to accumulate, to enter the nucleus and finally to interact with and repress the CLK/CYC complex. The result is a *circa* 24 h rhythm in *per* and *tim* mRNA and protein abundance, which, through mechanisms still not well understood, powers rhythmic physiology and behaviour. Two additional loops interlock with the first through the common element CLK. One involves the rhythmic expression of PAR DOMAIN PROTEIN 1ε (PDP1ε) and VRILLE (VRI), the other implicates the transcription factor CLOCKWORK ORANGE (CWO) (Blau and Young, 1999; Cyran et al., 2003; Lim et al., 2007; Richier et al., 2008). However, their dynamics are less understood (reviewed in Özkaya and Rosato, 2012). Finally, CRYPTOCHROME (CRY), a blue-light sensing protein, participates in the clock by mediating circadian and visual photoresponsiveness and light-dependent neuronal firing (Emery et al., 1998; Stanewsky et al., 1998; Fogle et al., 2011, 2015; Mazzotta et al., 2018; Schlichting et al., 2018). The concerted expression of these clock genes in lateral and dorsal neurons of the brain identifies the anatomical location of the clock (reviewed in Helfrich-Förster, 2003).

Here we explore the circadian system of *D. suzukii* in the laboratory. The motivation was to investigate whether this fly could become a convenient laboratory model for studying circadian rhythmicity in an invasive pest species that is phylogenetically quite close to *D. melanogaster* but differs in its ecological specialisations and in the tuning of its sensory systems. In particular, we wanted to assess whether in *D. suzukii*, we could record robust rhythmic behaviour, describe their neuronal clock network and putatively correlate behavioural and anatomical differences they might present compared to *D. melanogaster*. We intend this work as a prerequisite for future manipulations of the circadian system in *D. suzukii* using tools such as gene editing and transgenesis, with the ultimate goal of aiding the functional characterisation of the different clock neurons in both species. Thus, we tested rhythmic locomotor activity under several laboratory conditions and we examined the cellular expression of the clock genes PER, TIM, CRY, PDP1ε and of the clock-relevant neuropeptide PDF in the brain. We report large behavioural differences and some anatomical variations between the two species; these results form the basis for future investigations.

## Materials and Methods

### Fly Maintenance

Both species of flies were maintained in a light and temperature controlled room at LD 12:12, 25°C on yeast cornmeal media (water: 7,500 ml, maize meal: 504 g, glucose: 555 g, brewer's yeast: 350 g, agar: 59.5 g. Added after boiling: propionic acid: 21 ml, 20% nipagen in ethanol: 94.5 ml) in polystyrene growth vials (9 cm height x 2 cm diameter, Regina Industries, UK).

### Fly Strains

Strains of *D*. *suzukii* (S1202, S1203, S1209, S1210, SMichele) and *D. melanogaster* (M1206, M1217) were established from wild captured flies in the summer of 2012 near Trento in the North of Italy. Several gravid females were used to found each line.

### Locomotor Activity

#### Rearing

All flies were grown as above (Fly maintenance) irrespective of the entrainment regime they were going to be subject to when tested. Virgin females and males were collected on the day of eclosion and kept in small groups of 5–15 individuals (males only, females only or males and females, according to the experiment) into growth vials before loading into “activity tubes” (see below) and monitors. Flies were 4–7 days old when loaded. However, the population monitors were loaded on the same day of fly collection. In all but one experiment the flies were tested on the same yeast cornmeal media used for rearing. In the experiment where we compared males, virgin females and mated females, the flies (all types) were moved into an “activity tube” containing nitrogen-free medium (10% sucrose, 1.5% agar in water) on the day of loading.

#### Single-Fly Activity

Individual flies were loaded into a glass “activity tube” (5 mm in diameter × 80 mm in length, 1 mm in thickness) with medium at one end and a cotton plug at the other. The tubes were inserted (for a maximum of 32 channels) into an activity monitor (DAM2, Trikinetics, USA) that detects the breaking of an infrared beam (per channel) by the moving fly, sending the information to a computer for storage.

#### Population Rhythms

Ten males or 10 males and 10 females were placed in a growth vial after eclosion and then immediately loaded into a Drosophila Population monitor (DPM, Trikinetics, USA).

#### Rectangular Entrainment

All experiments using rectangular light-dark (LD) entrainment followed by constant darkness (DD) were carried out in LSM (UK) light and temperature controlled incubators equipped with three fluorescent tubes (TL-D 90 De Luxe 18W/940 SLV/10, Phillips, NL).

For every experiment 4 days in LD was followed by either 5 or 6 days in DD under constant temperature. In general we carried out experiments under LD12:12, 25°C and then DD. In one experiment we tested five entrainment conditions, followed by DD. The conditions were: L = long photoperiod (LD 16:8, 25°C), S = short photoperiod (LD 8:16, 25°C), H = hot temperature (LD 12:12, 28°C), C =cold temperature (LD 12:12, 18°C) and I = intermediate condition (LD 12:12, 25°C).

#### Seminatural Conditions

We used a programmable IPP500 Peltier incubator (Memmert, Germany) able to produce a precise temperature cycle that was custom modified to generate a smooth light cycle of desired spectral composition (see Green et al., 2015 for further information). We mimicked a midsummer's day in Northern Italy (where our flies had been collected) but exploring two temperature cycles, of 20–30°C (seminatural_1) or 25–35°C (seminatural_2). The day length was approximately LD 16:8 with a maximum light intensity of 350 lux and 300 lux, respectively for the two experiments. Note however that because of an error while programming the incubator *D. melanogaster* flies were exposed to a maximum of 250 lux during the first experiments while *D. suzukii* were exposed to 350 lux. In both experiments, the temperature cycle peaked 2.5 h later than the light cycle.

### Statistical Analyses

The circadian period of rhythmic locomotor activity was calculated using the CLEAN package (Rosato and Kyriacou, 2006). We used GraphPad Prism7 for all other statistical calculations and graphics. Descriptive statistics are reported in Tables and Figure legends.

### Immunofluorescence and Microscopy

Flies were entrained under LD 12:12, 25°C for more than 3 days. Flies were collected at ZT18 (anti-PDP1ε), at ZT11 and ZT23 (anti-PER and anti-TIM) and at ZT11 and (after 3 days in DD) at CT23 (anti-CRY). Whole flies were fixed for 2 h in 4% PFA with 0.1% Triton-X and 5% DMSO on a rotating wheel. Brains were dissected in 0.1% PBST (1xPBS with 0.1% Triton-X), permeabilized with 3 × 15 min washes in 1% PBST and blocked (either for 2 h at room temperature or 16 h at 4°C) in 0.5% PBST with added 5% goat serum. Primary antibodies were applied (in fresh blocking solution with added 0.1% Sodium Azide) for 4–6 days at 4°C. After 3 × 15 min washes in 0.5% PBST the brains were incubated with secondary antibodies (in PBST 0.5%) for 3 h. After 3 × 15 min washes in 0.5% PBST the brains were quickly rinsed in distilled water and incubated at 4°C in anti-fade solution (3% propyl gallate, 80% glycerol in PBS pH 8.5) overnight before mounting. Steps were carried out at room temperature unless specified.

Primary antibodies: anti-CRY 420753, rabbit (1:500; Dissel et al., 2014), anti-PDF C7, mouse (1:50; DSHB), anti-PER c-300, rabbit (1:50; Santa Cruz Biotech), anti-TIM UP991, rabbit (1:2000; Koh et al., 2006), anti-PDP1ε, rabbit (1:5000, Cyran et al., 2003).

Secondary antibodies: Anti-rabbit biotinylated (1:600) & streptavidin Dylight 649 (1:300) [used in conjunction with anti-CRY]; anti-mouse Cy2 (1:200); anti-rabbit Alexa 647 (1:200). All secondary antibodies were from Jackson ImmunoResearch. Images were captured with an Olympus FV100 confocal microscope.

## Results

### Locomotor Activity Under Standard LD Conditions

**Figure 1:**
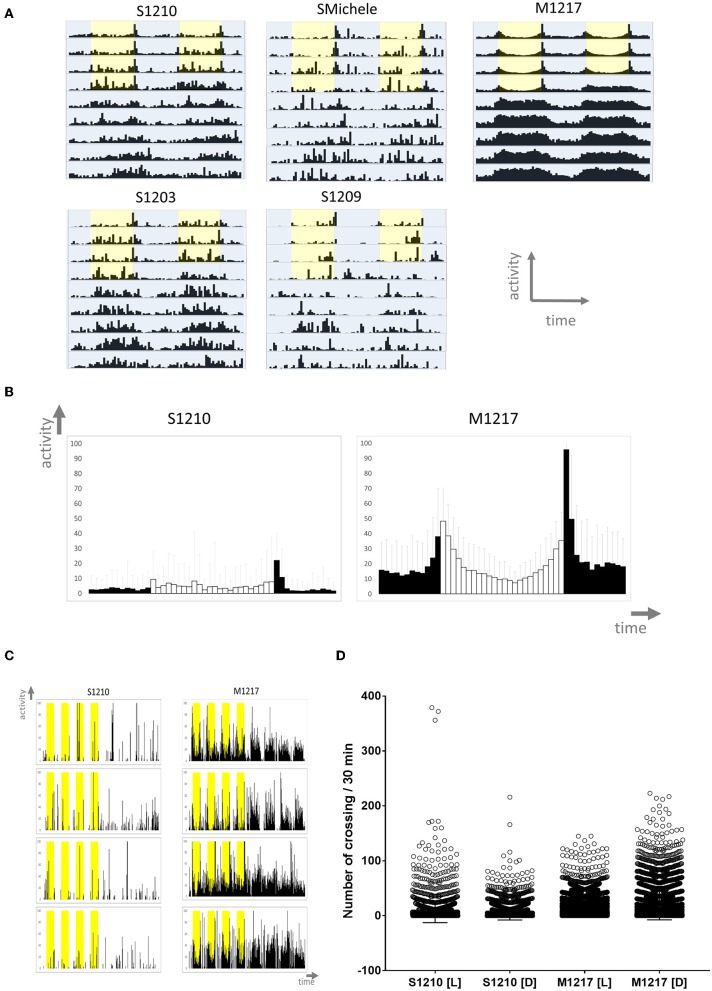
Locomotor activity in *D. suzukii* (S) and *D. melanogaster* (M) males under standard laboratory conditions. **(A)** Average locomotor activity profiles. Activity levels (number of crossing/30 min) are shown on the Y-axis; time (96 intervals of 30 min) is on the X-axis. Data are double plotted (i.e. day1-day2, day2-day3, etc.). Flies were monitored for 4 days under LD and for 6 days under DD at 25°C. Light is shown in yellow, darkness in blue highlight. Light-dark (LD) conditions followed a rectangular pattern (On-Off) until constant darkness (DD). To aid the visual appreciation of rhythmicity, activity levels are not to scale and standard deviation is omitted. Only rhythmic flies (both SR and CR, see Table 1) contributed to the graphs. **(B)** Average day profile for *D suzukii* S1210 and *D. melanogaster* M1217 males. The average day was obtained by combining into one the four LD days for all rhythmic flies (SR+CR, see Table 1) of the same genotype. The profile for *D. melanogaster* shows clear “Morning” and “Evening” peaks separated by a “siesta.” The profile for *D. suzukii* is not clearly defined. In general, it shows that the flies are most active during the light part of the day. Black columns correspond to dark and white columns to light. Error bars show standard deviation. The Y-axis shows activity levels (0–100 crossing/30 min); the X-axis shows time (48 intervals of 30 min). **(C)** Examples of activity profiles for individual flies. It is clear that *D. suzukii* are much less active than *D. melanogaster* flies and spend much of their time being inactive. During LD, *D. suzukii* are more active during the light portion of the day (yellow highlight). Conversely, *D. melanogaster* are crepuscular, being most active at the boundaries of the D to L and L to D transitions. The Y-axis shows activity levels (0–100 crossing/30 min); the X-axis shows time (384 intervals of 30 min). **(D)** Activity levels (number of crossing/30 min) for the L and D part of the day across the four LD days, have been plotted separately for rhythmic *D. suzukii* S1210 and *D. melanogaster* M1217 individual males. It is immediately evident that while in *D. suzukii* higher values are more common during light, the opposite is true for *D. melanogaster*. The four distributions are significantly different (Kruskal-Wallis, *P* < 0.0001) in all possible pairwise comparisons (Dunn's multiple comparisons test, *P* < 0.0001).

We started by analysing the locomotor activity behaviour of male flies under standard laboratory conditions. Male flies are usually the preferred subject of circadian studies as they can be assayed on regular fly medium without worrying about their mating status. Instead, mated females can only be tested using medium lacking a nitrogen source to impede the development of fecundated eggs; thus avoiding interference by the progeny. In the laboratory, locomotor activity is more commonly measured testing single flies. Typically the experiments are carried out at constant temperature under ‘rectangular' (i.e., on-off) 12 h light−12 h dark (LD 12:12) conditions for a few days followed by constant darkness (DD) for some more days (Rosato and Kyriacou, 2006). We collected *D. suzukii* flies from the wild in 2012 near Trento (Italy) and used several gravid females to found each strain. In parallel we collected *D. melanogaster* flies also and we founded several strains in the same way. Initially we investigated four *D. suzukii* strains (S1210, SMichele, S1203 and S1209) and we compared them to one *D. melanogaster* line (M1217). We monitored male flies for 4 days under LD 12:12, and then for 6 days under DD, at the constant temperature of 25°C. As expected, *D. melanogaster* wild type M1217 flies survived the experimental conditions well (about 10% died before the end of the experiment) and were very active. They showed a bimodal pattern of activity (a Morning and an Evening peak in correspondence to the D/L and L/D transitions) during LD, and were highly rhythmic under DD (Figure 1A and Table 1). *D. suzukii* did not perform as well under the same conditions. A considerable percentage of flies (from 30% to almost 60% according to strain, Table 1) did not survive until the end of the experiment, and those that did, showed a much lower level of activity than *D. melanogaster*. This can be appreciated in Figures 1B,C (instead Figure 1A is not to scale) showing, for S1210 and M1217, the average day activity profile for all rhythmic flies and representative individual activity profiles, respectively. From the figures it is also clear that S1210 flies are rather more diurnal than bimodal. To confirm this observation, we compared the distribution of activity (the number of crossing of the infrared beam for every 30 min interval—“bin”—per fly) under light (L) and dark (D) conditions between the two species. Figure 1D shows that for S1210 flies, “bins” with higher activity are more common during L than D, whereas the opposite is true for M1217, in agreement with the expectations for a crepuscular species. Figure 1A and Figure S1 qualitatively confirm that in *D. suzukii* a “noisy” M peak, if present, is found at the end of the morning, and that a “noisy” E peak tends to eclipse after lights off. Additionally, we noticed that all *D. suzukii* strains showed a very high percentage of arrhythmic flies under DD conditions, and a large variance in period among those that were rhythmic. This contrasts with higher rhythmicity and lower period variance, which we observed for *D. melanogaster* (Table 1).

**Table 1:** Locomotor activity statistics of five *D. suzukii* lines in comparison with one *D. melanogaster* line established from flies collected in parallel in the wild.

**Species**	**Line**	**Gender**	**NT**	**D**	**D (%)**	**N**	**SR**	**SR (%)**	**CR**	**CR (%)**	**AR**	**AR (%)**	**τ [SR]**	**Sdev [SR]**
Ds	S1210	m	185	75	41	110	44	40	0	0	66	60	24.52	3.12
Ds	S1209	m	64	34	53	30	6	20	2	7	22	73	23.78	1.6
Ds	SMichele	m	64	38	59	26	6	23	2	8	18	69	25.27	1.24
Ds	S1203	m	64	21	33	43	16	37	2	5	25	58	24.63	1.79
Dm	M1217	m	48	5	10	43	41	96	1	2	1	2	24.26	0.93

*Ds, D.suzukii; Dm, D. melanogaster; m, males; NT, total number of flies tested; D, number of flies that died before the end of the 6th day in DD. These have been excluded from further analysis. D (%), D/NTx100; N, number of flies that survived the whole experiment; SR, flies showing a single rhythm of activity in free run; SR(%), SR/Nx100; CR, flies showing more than one (complex) activity period in free run; CR(%), CR/Nx100; AR, arrhythmic flies; AR(%), AR/Nx100; τ [SR], circadian period of locomotor activity of SR flies; Sdev [SR], standard deviation of the circadian period of SR flies*.

### Temperature Compensation and Entrainment

We then decided to test two fundamental properties of the clock, temperature compensation and entrainment.

**Figure 2:**
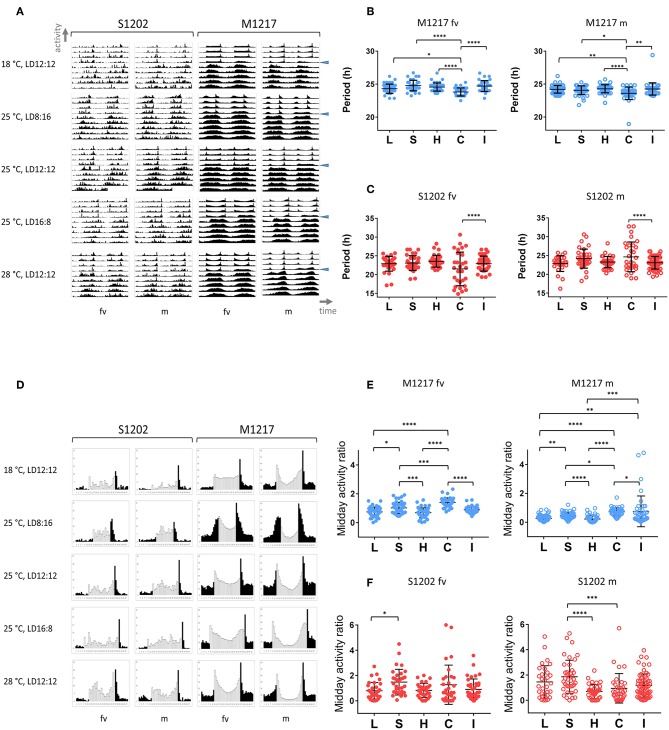
Locomotor activity in *D. suzukii* and *D. melanogaster* virgin females (fv) and males (m) under different entrainment regimes: L, LD 16:8, 25°C (long photoperiod); S, LD 8:16, 25°C (short photoperiod); H, LD 12:12, 28°C (hot temperature); C, LD 12:12, 18°C (cold temperature); I, LD 12:12, 25°C (intermediate condition). **(A)** Average locomotor activity profiles. Activity levels (number of crossing/30 min) are shown on the Y-axis; time (96 intervals of 30 min) is on the X-axis. Data are double plotted (i.e. day1-day2, day2-day3, etc.). Flies were monitored for 4 days under LD and for 6 days under DD. The beginning of DD is shown by a blue arrow head. To aid the visual appreciation of rhythmicity, L and D are not highlighted, activity levels are not to scale and standard deviation is omitted. Only rhythmic flies (both SR and CR, see Table 2) contributed to the graphs. **(B)** Distribution of circadian locomotor activity periods (SR flies only, see Table 2) in *D. melanogaster* following different entrainment regimes. Note that we report free run periods but for ease of description the labelling refers to the preceding entrainment conditions. Virgin females are on the left, males are on the right. Periods were compared across all conditions (Kruskal-Wallis, *P* < 0.0001 for both virgin females and males). The significant difference is explained by a shorter period under C (cold temperature, LD 12:12, 18°C) compared to all other conditions (Dunn's multiple comparisons tests, *P < 0.05; **P < 0.01; ****P < 0.0001). However, this does not violate the principle of temperature compensation (see text). **(C)** Distribution of circadian locomotor activity periods (SR flies only, see Table 2) in *D. suzukii* following different entrainment regimes. Note that we report free run periods but for ease of description the labelling refers to the preceding entrainment conditions. Virgin females are on the left, males are on the right. For each group, comparing periods across all conditions did not result in a significant difference by Kruskal-Wallis test. However, for both males and virgin females there was a significant increase in the variance when locomotor activity was recorded under the cold temperature condition (F test, C vs. I, *P* < 0.0001). This suggests that under these experimental conditions *D. suzukii* might lose temperature compensation at lower temperatures. **(D)** Average day profile for *D suzukii* S1202 and *D. melanogaster* M1217 virgin females (fv) and males (m). The average day was obtained as for Figure 1B. Black columns correspond to dark and white columns to light. For ease of visualisation the standard deviation is not reported. The Y-axis reports activity levels: *D. suzukii* 0-25 (crossing/30 min), *D.melanogaster* 0-60 (crossing/30 min). The X-axis reports time (48 intervals of 30 min). Note that for M1217 m, LD12:12, 25°C, the figure is the same as Figure 1B. **(E)** Midday activity ratio (rhythmic flies only, both SR and CR, see Table 2) in *D. melanogaster* under different entrainment regimes. The midday activity ratio is a measure of how much a fly is active (on average) in the middle part of the light-phase (the three central hours of the light portion of the day) relative to the average activity across the whole 4 days in LD. Values closer to zero indicate a robust siesta, values higher than one indicate that the fly is more active than average in the middle of the day. Virgin females are on the left, males are on the right. Both virgin females and males showed increased midday activity at lower temperature (C) and shorter photoperiod (S), whereas more siesta occurs under higher temperature (H) and longer photoperiod (L). Significant differences are confirmed overall by Kruskal-Wallis (*P* < 0.0001 for both genders) and between conditions by the Dunn's multiple comparison test (*P < 0.05; **P < 0.01; ***P < 0.001; ****P < 0.0001) **(F)**. Midday activity ratio (rhythmic flies only, both SR and CR, see Table 2) in *D. suzukii* under different entrainment regimes. Virgin females are on the left, males are on the right. For both genders the responses were highly variable. For virgin females there was a significant difference among distributions (Kruskal-Wallis, *P* < 0.05). Although we could observe a tendency for more activity in the middle of the day at lower temperature (C, LD 12:12, 18°C) and under short photoperiod (S, LD 8:16, 25°C) compared to other conditions, only the comparison between the two extreme photoperiods (L and S) maintained significance under a multiple comparison test (Dunn's, L vs. S, *P* < 0.05). We observed a significant difference among distributions also for males (Kruskal-Wallis, *P* < 0.0001). However, the situation was less clear as we noticed a relative increase in siesta not only at the higher (H, LD 12:12, 28°C) but also at the lower (C, LD 12:12, 18°C) temperature. Both conditions were different with respect to the short photoperiod under a multiple comparison test (Dunn's; C vs. S, *P* < 0.001; H vs. S, *P* < 0.0001).

In general chemical and biochemical reactions change proportionally to temperature. Temperature compensation refers to the ability of the clock to withstand relatively large variations in temperature while only limited changes in period occur. This is an adaptive property of the clock and is subject to scrutiny by natural selection (Pittendrigh, 1993; Sawyer et al., 1997). We compared *D. melanogaster* M1217 to *D. suzukii* S1202. The latter was chosen because its behaviour is similar to S1203 and S1210 but is more prolific. To avoid the confounding influence of possible aftereffects that might arise due to exposure to different environmental conditions during development, we raised all flies under LD 12:12, 25°C. Then, we monitored virgin females (fv) and males (m) for 4 days under five entrainment conditions followed by 6 days under DD (free run). For each fly we calculated the circadian period of locomotor activity; namely, each period derives from the free run part of the experiment and represents the endogenous rhythmicity of the fly. However, to simplify the explanation, here we refer to the entrainment condition that preceded the free run when describing the period results. The five conditions were: L = long photoperiod (LD 16:8, 25°C), S = short photoperiod (LD 8:16, 25°C), H = hot temperature (LD 12:12, 28°C), C = cold temperature (LD 12:12, 18°C) and I = intermediate condition (LD 12:12, 25°C). Figure 2A provides a snapshot of the average locomotor activity profiles under all conditions; Table 2 shows the descriptive statistics. For M1217, Figure 2B and Table 2 show that the cold temperature (C, LD 12:12, 18°C) is the only condition resulting in a small but significant shortening of the circadian period for both virgin females (−0.75 h compared to H, LD 12:12, 28°C) and males (−0.68 h compared to H, LD 12:12, 28°C). Additionally, in all conditions and for both genders we observed substantially the same variance. These results are in line with previous observations for the species and confirm that *D. melanogaster* is capable of robust temperature compensation in the interval 18–28°C (Sawyer et al., 1997). The situation for S1202 was different (Figure 2C and Table 2). Both virgin females and males showed changes in circadian period across conditions but these failed to reach statistical significance. However, for both genders the cold temperature condition (LD 12:12, 18°C) showed a characteristic larger variance, which was significantly different (F test, *P* < 0.0001 for both genders) when compared to the intermediate condition (LD 12:12, 25°C) (Figure 2C). As a result, in the cold temperature condition (LD 12:12, 18°C) both genders showed a difference in circadian period of about 10–15 h between individuals at the extremes of the distribution. This suggests that in the laboratory, under cooler conditions, temperature compensation may be compromised in this species. This result is unexpected, not only because temperature compensation is a fundamental property of a functioning clock (Pittendrigh, 1993) but also because a temperature of 18°C should be a good match for a species that evolved in a temperate climate (Ometto et al., 2013). A caveat is that we only tested one line under these conditions and it may not be representative of the whole species.

**Table 2:** Locomotor activity statistics in *D. suzukii* (S1202) and *D. melanogaster* (M1217) males and virgin females under different entrainment conditions.

**Condition**	**Line**	**Gender**	**NT**	**D**	**D (%)**	**N**	**SR**	**SR (%)**	**CR**	**CR (%)**	**AR**	**AR (%)**	**τ [SR]**	**Sdev [SR]**
L	S1202	fv	86	18	21	68	32	47	1	1	35	52	22.90	1.97
S	S1202	fv	96	28	29	68	32	47	0	0	36	53	23.07	1.96
H	S1202	fv	96	44	46	52	33	63	0	0	19	37	23.55	1.58
C	S1202	fv	96	8	8	88	29	33	2	2	57	65	21.51	4.47
I	S1202	fv	125	34	27	91	35	39	3	3	53	58	22.92	2.01
L	S1202	m	90	15	17	75	27	36	4	5	44	59	22.85	2.09
S	S1202	m	96	29	30	67	37	55	3	5	27	40	24.17	2.53
H	S1202	m	96	33	34	63	36	57	0	0	27	43	23.26	1.49
C	S1202	m	96	12	13	84	30	36	3	3	51	61	24.64	3.96
I	S1202	m	127	15	12	112	60	54	3	2	49	44	23.08	1.66
L	M1217	fv	54	4	7	50	42	84	5	10	3	6	24.31	0.70
S	M1217	fv	48	3	6	45	37	82	7	16	1	2	24.79	0.80
H	M1217	fv	48	3	6	45	42	93	3	7	0	0	24.56	0.62
C	M1217	fv	48	2	4	46	39	85	1	2	6	13	23.81	0.61
I	M1217	fv	40	3	8	37	30	81	5	14	2	5	24.71	0.83
L	M1217	m	58	0	0	58	54	93	0	0	4	7	24.21	0.58
S	M1217	m	48	1	2	47	45	96	1	2	1	2	24.07	0.67
H	M1217	m	48	0	0	48	44	92	1	2	3	6	24.28	0.61
C	M1217	m	48	0	0	48	39	81	2	4	7	15	23.60	0.95
I	M1217	m*	48	5	10	43	41	96	1	2	1	2	24.26	0.93

L, long photoperiod (LD 16:8, 25°C); S, short photoperiod (LD 8:16, 25°C); H, hot temperature (LD 12:12, 28°C); C, cold temperature (LD 12:12, 18°C); I, intermediate conditions (LD 12:12, 25°C); m, males; fv, virgin females; NT, total number of flies tested; D, number of flies that died before the end of the 6th day in DD. These have been excluded from further analysis. D (%), D/NTx100; N, number of flies that survived the whole experiment; SR, flies showing a single rhythm of activity in free run; SR(%), SR/Nx100; CR, flies showing more than one (complex) activity period in free run; CR(%), CR/Nx100; AR, arrhythmic flies; AR(%), AR/Nx100; τ [SR], circadian period of locomotor activity of SR flies; Sdev [SR], standard deviation of the circadian period of SR flies. [*] These are the same as in Table 1.

Entrainment refers to the ability of the clock to synchronise to and “remember” external rhythmic stimuli or *Zeitgebers* (German for “time givers”). One important consequence of entrainment is the fact that circadian rhythms adopt a defined phase relationship with the external cycles, resulting in time-specific allocation of activities during the day. Figure 2D shows the average day for S1202 and M1217 virgin females and males. Turning our attention to *D. melanogaster* (M1217) males first, we can qualitatively appreciate that the “siesta” (the interval of reduced activity in between the M and E peaks) is more pronounced at higher temperatures (siesta_28∙C_ > siesta_25∙C_ > siesta_18∙C_) or under longer photoperiods (siesta_LD16:8_ > siesta_LD12:12_ > siesta _LD8:16_). To quantify such an effect we calculated for each fly a “midday activity ratio” under all conditions. This is the ratio between the average activity during the three central hours of the light phase and the average activity across the whole LD period. Figure 2E shows that such a variable was significantly different across conditions (Kruskal-Wallis *P* < 0.0001) and maintained significance between many pairwise comparisons (Dunn's multiple comparison test). Likewise, *D. melanogaster* virgin females showed a more pronounced “siesta” at higher temperatures or under longer photoperiods (Figure 2D), which was further confirmed by comparing the “midday activity ratio” across conditions (Figure 2E). However, *D. melanogaster* virgin females showed less “siesta” compared to males under all conditions (Figure 2D), which was in agreement with an overall higher level of activity (Figure S2). Gender differences in locomotor activity are well documented in *D. melanogaster* (Helfrich-Förster, 2000).

Conversely, we could not identify clear-cut differences among conditions and between genders for *D. suzukii*. Inspection of Figure 2D does not reveal prominent M and E peaks and “siesta” with the exception of the hot temperature condition (LD 12:12, 28°C). In addition, there were no striking gender differences. In general, males showed an increase in activity towards the end of the morning resulting in a modest M peak. Such a peak appeared earlier (LD 12:12 and LD 16:8, 25°C) or was absent (LD 8:16, 25°C) in females, except in the hot condition (LD 12:12, 28°C). However, we did not find an overall difference in activity levels between genders (Figure S2). Finally, we compared the “midday activity ratio” among the males and the females (Figure 2F). In both genders we observed an overall difference (Kruskal-Wallis, males *P* < 0.0001, virgin females *P* < 0.05). Both males and virgin females showed a general increase of the “midday activity ratio” (i.e., more activity in the middle of the day) under short photoperiod (LD 8:16, 25°C) and a reduction (less activity in the middle of the day) under the hot condition (LD 12:12, 28°C). *Post-hoc* (Dun's multiple comparison) tests revealed a significant difference between short (LD 8:16, 25°C) and long (LD 16:8, 25°C) photoperiod for virgin females (P <0.05). For males, two comparisons, short (LD 8:16, 25°C) vs. hot (LD 12:12, 28°C) and short (LD 8:16, 25°C) vs. cold (LD 12:12, 18°C) maintained significance (*P* < 0.0001 and *P* < 0.001, respectively). For the latter, an inspection of the average day profile (Figure 2D) revealed that under the cold condition (LD 12:12, 18°C) males showed a dramatic reduction in activity during the whole light portion of the LD cycle (not just the middle of the day), whereas the “startle” increase of activity at the L/D switch was still prominent. This highlights a potential difference between genders in *D. suzukii*.

### The Effect of Mating on Females' Activity

**Figure 3:**
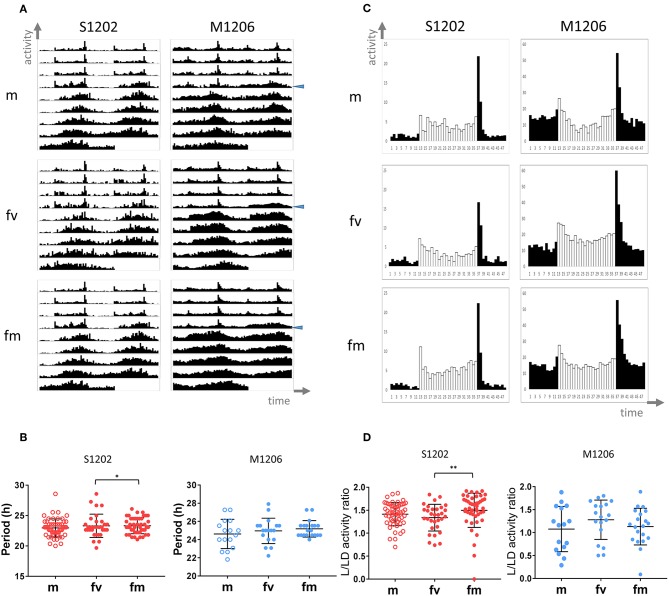
Locomotor activity in *D. suzukii* and *D. melanogaster* males (m), virgin (fv) and mated (fm) females under standard laboratory conditions. **(A)** Average locomotor activity profiles. Activity levels (number of crossing/30 min) are shown on the Y-axis, time (96 intervals of 30 min) is on the X-axis. Data are double plotted (i.e. day1-day2, day2-day3, etc.). Flies were monitored for 4 days under LD 12:12 and for 5 days under DD at 25°C. The beginning of DD is shown by a blue arrow head. To aid the visual appreciation of rhythmicity L and D are not highlighted, activity levels are not to scale and standard deviation is omitted. Only rhythmic flies (both SR and CR, see Table 3) contributed to the graphs. **(B)** Circadian period of locomotor activity (SR flies only, see Table 3) in *D. suzukii* (left) and *D. melanogaster* (right). For each species, comparing circadian periods among males (m) virgin females (fv) and mated females (fm) did not result in a significant difference by Kruskal-Wallis test. However, both species showed reduced variance in mated *vs*. virgin females; for *D suzukii* such a difference was significant (F test, *P < 0.05). **(C)** Average day profile for *D suzukii* S1210 and *D. melanogaster* M1206 males (m), virgin females (fv) and mated females (fm). The average day was obtained as for Figure 1B. Black columns correspond to dark and white columns to light. For ease of visualisation the standard deviation is not reported. The Y-axis reports activity levels: *D. suzukii* 0–25 (crossing/30 min), *D. melanogaster* 0–60 (crossing/30 min). The X-axis reports time (48 intervals of 30 min). **(D)** L/LD activity ratio (rhythmic flies only, both SR and CR, see Table 3) in *D. suzukii* (left) and *D. melanogaster* (right) males (m), virgin females (fv) and mated females (fm). The L/LD activity ratio is a measure of how much a fly is active (on average) during the L part of the LD cycle relative to the average activity across the whole 4 days in LD. Values closer to zero indicate a reduction of activity during L whereas values higher than one indicate more activity during L than the LD average. Both species showed large variability in the response. In *D. suzukii* the three distributions were significantly different (Kruskal-Wallis, *P* < 0.01). Dunn's multiple comparison tests confirmed an increase in activity during the light-phase in mated vs. virgin females (**P < 0.01). In *D. melanogaster* the three distributions were not statistically different.

In *D. melanogaster* females, mating causes an increase in activity during the light phase of the LD cycle (Isaac et al., 2010). To investigate whether mating affects *D. suzukii* also, we analysed the locomotor activity of S1202 using *D. melanogaster* M1206 as a comparison (due to a technical problem we did not obtain sufficient M1217 flies in this occasion). We tested males (m), virgin (fv) and mated females (fm) on a nitrogen-free medium at 25°C. Figure 3A shows the average locomotor activity profiles (not to scale) under LD 12:12 (first 4 days) and then DD (following 5 days). Table 3 reports the descriptive statistics. First we analysed the period of rhythmic locomotor activity under DD (Figure 3B). In both species, differences in circadian period were not significant across the three groups (Kruskal-Wallis). However, mating resulted in reduced variance in the circadian period of females, which was significant for S1202 (F test, *P* < 0.05; Figure 3B). To compare activity under the light-phase of the LD cycle, we first derived the average day profiles (Figure 3C). Visual inspection did not reveal any obvious difference between *D. melanogaster* virgin and mated females but a possible increase in activity during the light phase for *D. suzukii* mated females compared to virgins. To quantify such a difference we calculated for each rhythmic fly the L/LD activity ratio, which measures the average relative activity under light with respect to the whole LD cycle (Figure 3D). For *D. melanogaster* we did not observe significant changes, but for *D. suzukii* the L/LD activity ratio was different when compared across the three conditions (Kruskal-Wallis, *P* < 0.01). *Post-hoc* analyses confirmed an increase in activity during the light phase in mated vs. virgin females (Dunn's multiple comparison test, *P* < 0.01). Possibly, small sample size and/or genetic background could explain the lack of difference across the three conditions for M1206.

**Table 3:** Locomotor activity statistics in *D. suzukii* (S1202) and *D. melanogaster* (M1206) males, virgin and mated females.

**Line**	**Gender**	**NT**	**D**	**D (%)**	**N**	**SR**	**SR (%)**	**CR**	**CR (%)**	**AR**	**AR (%)**	**τ [SR]**	**Sdev [SR]**
S1202	m	128	26	20	102	54	53	1	1	47	46	22.96	1.49
S1202	fv	120	55	46	65	31	48	0	0	34	52	23.31	1.92
S1202	fm	119	45	38	74	46	62	0	0	28	38	23.31	1.31
M1206	m	32	13	41	19	16	84	0	0	3	16	24.62	1.61
M1206	fv	32	5	16	27	18	67	0	0	9	33	24.96	1.40
M1206	fm	32	7	22	25	20	80	0	0	5	20	25.19	0.91

*M, males; fv, virgin females; fm, mated females; NT, total number of flies tested; D, number of flies that died before the end of the 5th day in DD. These have been excluded from further analysis. D (%), D/NTx100; N, number of flies that survived the whole experiment; SR, flies showing a single rhythm of activity in free run; SR(%), SR/Nx100; CR, flies showing more than one (complex) activity period in free run; CR(%), CR/Nx100, AR, arrhythmic flies; AR(%), AR/Nx100; τ [SR], circadian period of locomotor activity of SR flies; Sdev [SR], standard deviation of the circadian period of SR flies*.

### Seminatural Conditions

The main difficulty in recording locomotor activity in *D. suzukii* flies lies in their inactivity. We wondered whether we could elicit higher locomotion by mimicking conditions that are closer to the natural environment. For instance, in nature both light and temperature change gradually, with temperature lagging few hours behind light. Similar conditions, dubbed seminatural, can be reproduced in the laboratory with the aid of sophisticated incubators as described by Green et al. (2015). We used the same equipment and compared S1202 and M1217 males.

**Figure 4:**
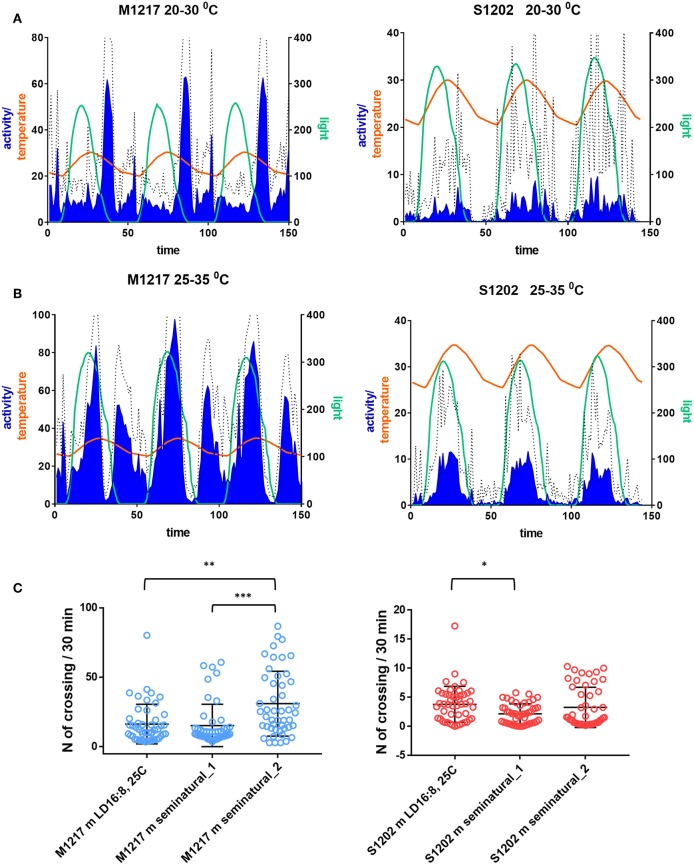
Locomotor activity in *D. suzukii* (S1202) and *D. melanogaster* (M1217) males under seminatural conditions. Flies were exposed to cycling temperature and to cycling light of spectral composition mimicking a midsummer's day in Northern Italy. The temperature cycle peaked 2.5 h later than the light cycle. Only flies that survived in excess of 3 days were considered. **(A)** Seminatural condition 1. Left Y-axis: Activity levels (blue), *D. suzukii* 0–40 (crossing/30 min), *D. melanogaster* 0–80 (crossing/30 min). Temperature (orange), 20–30°C. Right Y-axis: Light (green), *D. suzukii* 0–350 lux, *D. melanogaster* 0–250 lux. X-axis: Time (150 intervals of 30 min). Hatched line: standard deviation of activity. S1202, *N* = 66/76. M1217, *N* = 32/32. [*N* = alive after 3 days/initial number]. **(B)** Seminatural condition 2. Left Y-axis: Activity levels (blue), *D. suzukii* 0–40 (crossing/30 min), *D. melanogaster* 0–100 (crossing/30 min). Temperature (orange), 25–35°C. Right Y-axis: Light (green, 0–300 lux. X-axis: Time (150 intervals of 30 min). Hatched line: standard deviation of activity. S1202, *N* = 47/107. M1217, *N* = 30/30. **(C)** Average day activity levels (number of crossing/30 min) of LD 16:8, 25°C, seminatural conditions 1 and 2. Activity levels were different in M1217 males under the three conditions (Kruskal-Wallis, *P* < 0.0001). Dunn's multiple comparisons test confirmed an increase in activity under seminatural conditions 2 (LD16:8, 25°C vs. seminatural_2, **P = 0.0021; seminatural_1 vs. seminatural_2, ***P = 0.0001). Activity levels were different in S1202 males under the three conditions (Kruskal-Wallis, *P* = 0.0317). Dunn's multiple comparisons test confirmed a small decrease in activity of seminatural conditions 1 compared to LD16:8, 25°C (*P = 0.028).

In the first set of experiments S1202 males were subject to cycling light between 0–350 lux *ca*. (of spectral composition mimicking natural midsummer light in Northern Italy) and cycling temperature between 20 and 30°C (set to reach its maximum 2.5 h later than the light peak, see Green et al., 2015). The photoperiod was about LD 16:8. The M1217 *D. melanogaster* comparison was meant to be set to the same conditions but an error while programming the incubator resulted in a lower amplitude of the light cycle (0–250 lux *ca*.), the other parameters were the same. Figure 4A shows the average activity profile for the two species across three days, after which the mortality rate for *D. suzukii* became too high (especially during the second set of experiments). Under those conditions M1217 males substantially reduced their activity when the temperature started rising and increased their activity again in the last portion of the light-phase when the temperature began falling. The main peak of activity was in the early night followed by a short rest phase and then by a smaller bout of activity just before the lights came on corresponding to the coolest part of the day. A small increase in activity followed the beginning of light but it was quickly curbed by the rising of temperature. The complexity of this activity profile shows that in *D. melanogaster* locomotion is not regulated by a permissive/inhibitory temperature or light threshold; in fact, at the same absolute values of temperature and light flies could be mainly active or inactive depending on whether light and temperature were falling or rising. Thus, a quite sophisticated laboratory setting shows that isolated *D. melanogaster* flies are able to integrate sensory modalities and interpret their dynamic patterns to modulate locomotor activity rhythms. If we compare the profile of activity obtained under gradual changes in light and temperature and that obtained under a rectangular on-off LD 16:8 at constant 25°C (Figure 2D), the main difference is that under seminatural conditions the flies became primarily nocturnal as the morning and evening peaks of activity were confined to the end and the beginning of the cooling phase, respectively. Unlike the distribution, the amount of activity did not change dramatically, as shown in Figure 4C where we compare the amount of activity per “bin” of the average day. Conversely, under the first set of seminatural conditions, *D. suzukii* males were active almost exclusively during the light phase. Moreover, individuals were highly variable in their locomotor behaviour, as shown by the large standard deviation (Figure 4A). In comparison to the LD 16:8, 25°C condition (Figure 2D), the flies did not seem to organise their activity differently across the 24 h (a part from losing the startle response at the L to D transition) but they reduced its amount, although by a modest degree (Figure 4C).

In a second set of experiments both species were challenged with warmer conditions. The temperature cycled between 25 and 35°C, lagging 2.5 h behind a 0-300 lux *ca*. light cycle. In agreement with previous reports (Vanin et al., 2012; Green et al., 2015), *D. melanogaster* males became extremely active during the day with a large bout in the early afternoon. After a rest during the last part of the light cycle, activity started again in darkness. This time the evening and the morning peaks converged into a single rhythmic episode that terminated at the end of each cooling phase (Figure 4B). Overall this resulted in a large increase in activity (Kruskal-Wallis test, *P* < 0.0001. Dunn's multiple comparisons test: “seminatural_2” vs. “LD16:8, 25°C”, *P* = 0.0021; “seminatural_2” vs. “seminatural_1”, *P* = 0.0001. Figure 4C). The afternoon peak has been interpreted as a stress response that is modulated by the circadian clock and requires the thermo-sensitive channel transient receptor potential A1 (TrpA1) (Menegazzi et al., 2012; Green et al., 2015). Under this second set of seminatural conditions *D. suzukii* males became even more diurnal (Figure 4B). They showed a likely equivalent of the afternoon peak but no activity at other times. Thus, their total activity did not change substantially compared to rectangular LD 16:8, 25°C and to the first seminatural settings (Figure 4C).

Overall these experiments further confirm that in the laboratory *D. suzukii* are predominantly diurnal, in contrast to the crepuscular behaviour shown by *D. melanogaster*. Moreover, they highlight the different responses of the two species. While *D. melanogaster* flies are able to reallocate their activity to extend more into the day or the night according to the environmental conditions, *D. suzukii* seem to have just one temporal modality constantly tuned to “diurnal.” In addition, they seem to be much less active especially under stress and perhaps they employ immobility (saving energy and resources) as a general strategy to overcome unfavourable conditions. Anecdotally, a startling stimulus causes different levels of arousal in the two species (see Video 1).

### Synchronisation at the Population Level

**Figure 5:**
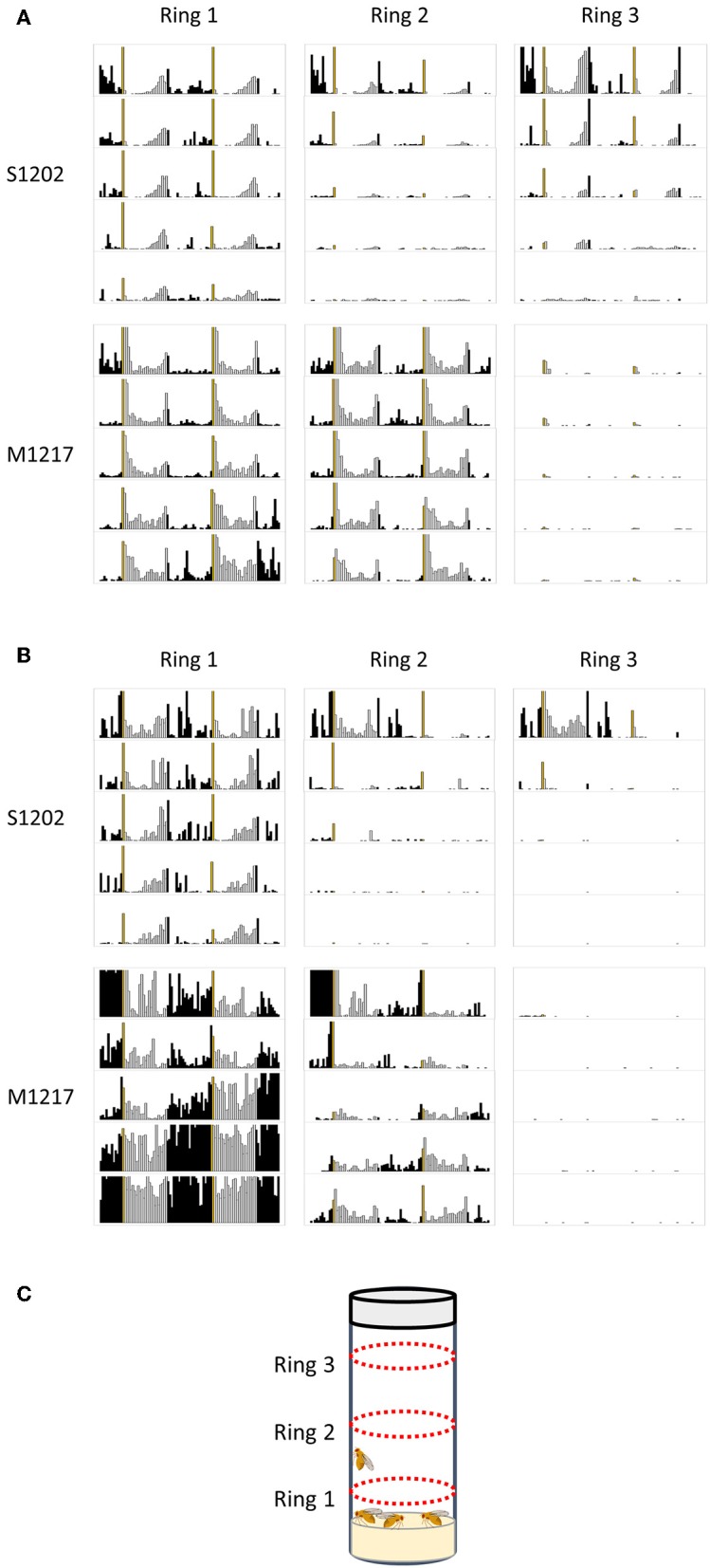
Population rhythms in *D. suzukii* (S1202) and *D. melanogaster* (M1217). **(A)** Ten males monitored together. **(B)** Ten males and ten females monitored together. Note that for M1217 the high levels of activity detected by Ring1 after 3 days are probably caused by the development of wandering larvae (see text). **(C)** Schematic representation of the experimental set-up showing the position of the three emitters/detectors Rings. Black columns correspond to dark and white columns to light. The yellow column corresponds to lights on. The Y-axis reports activity levels: 0–300 (crossing/30 min). The X-axis reports time (96 intervals of 30 min). Data are double plotted (i.e. day1-day2, day2-day3, etc.). Flies were monitored for 5 days in a growth vial (9 cm height x 2 cm diameter, with 1.5 cm of standard fly medium at the bottom and 1.5 cm stopper at the top) loaded into a *Drosophila* population monitor (DPM, Trikinetics, USA) under LD 12:12, 25°C. The DPM has three rings of infrared emitters/detectors; Ring1 was located 0.5 cm above the food, Ring 2 in the middle of the tube (3 cm above the food) and Ring 3, 0.5 cm below the stopper.

If our hypothesis is correct and *D. suzukii* flies reduce or shut down activity in response to stress, we would expect that monitoring locomotion under more favourable conditions would result in higher activity and better rhythmicity. We carried out pilot experiments under LD 12:12, 25°C. We tested the locomotor behaviour of 10 males kept together (Figure 5A) or 10 males and 10 females kept together (Figure 5B) in a growth vial (9 cm height x 2 cm diameter, with 1.5 cm of standard fly medium at the bottom and 1.5 cm stopper at the top), employing a population monitor (DPM, Trikinetics, USA). The DPM has three rings of infrared emitters/detectors; Ring1 (R1) was located 0.5 cm above the food, Ring 2 (R2) in the middle of the tube (3 cm above the food) and Ring 3 (R3), 0.5 cm below the stopper (Figure 5C). Figure 5A shows the average activity profiles (to scale) for ten S1202 and M1217 males. *D. suzukii* males spent most of the time near the food (R1) or at the other end of the vial (R3). The flies were active mostly in the evening and showed a consistent E peak anticipating the L/D transition, although an additional, small activity bout was present towards the end of the dark period. The flies were at rest in the morning, apart from a startle response in correspondence to the D/L transition. This contrasts with the average profile of single flies under similar LD and temperature conditions, showing a more disperse pattern of activity with poorly defined M and E peaks and no activity in the dark (Figures 1B, 2D). Moreover, *D. suzukii* males were much more active when monitored together than in isolation. Although a direct comparison between the two conditions is not possible, we can compare the relative activities of D. *suzukii* and *D. melanogaster* flies under the same conditions. When monitored individually S1202 males were about five times less active than M1217 males (Figure S2). When monitored as a group of males the combined activity (R1-R3) of S1202 flies was about 70% that of M1217 (Figure S3 and Table S1). Differences in activity profile when monitoring single males or group of males were also observed for *D. melanogaster*. Under group monitoring, the morning peak was more prominent but the siesta was less defined than for single male recordings (compare Figures 2D, 5A). Interestingly, most of the activity was recorded by R1 and R2, which again sets *D. melanogaster* apart from *D. suzukii*.

We then measured the locomotor activity of a group of 10 males and 10 females housed together (Figure 5B). For S1202 we observed an increase in morning and (especially) night activity, although the E peak was still the most prominent. We noticed that flies were mostly active near the food (R1), which we interpret as a consequence of egg-laying. Likewise, M1217 flies were much more active at night and additionally in the morning. Again, R1 recorded the majority of the activity. We saw a dramatic increment in R1 activity at the end of day 3, which we attribute to the development of wandering larvae. Our assumption is compatible with the timing of development of *D. melanogaster* and with the fact that such an increase in activity is limited to R1. The reason we did not observe a similar phenomenon for *D. suzukii* might be related to a longer developmental time for the species (Asplen et al., 2015) and to differences in larval behaviour, but we did not investigate this further.

Overall, these observations suggest that social interactions contribute to entrainment, as they refine the phase relation between rhythmic locomotor activity and the LD cycle. This was already known in *D. melanogaster* (Levine et al., 2002; Fujii et al., 2007). Additionally our data suggest that for species such as *D. suzukii*, enriched social conditions might be necessary for meaningful circadian behaviour to become manifest.

### The Neuronal Organisation of the Clock

The anatomical location of the clock is identified by the expression of clock genes and proteins. In *D. melanogaster* the clock consists of about 75 neurons per brain hemisphere, which are divided into lateral and dorsal neurons. The lateral neurons are then subdivided into ventral (LNv) and dorsal (LNd). The LNv consists of 4 large (l-LNv) and 4 small (s-LNv) neurons, both expressing the neuropeptide PIGMENT-DISPERSING FACTOR or PDF. Additionally, a single, PDF-null (pn-) or 5th-LNv is interspersed among the l-LNv. All LNv express CRY at high levels. More dorsal are 6 LNd. Of these, 3 express CRY at high levels whereas the others express *cry* mRNA but the protein is almost undetectable. Dorsal and posterior to the LN are the dorsal neurons, DN. These are divided into three heterogeneous groups, DN1, DN2 and DN3. In the DN1 cluster the 2 more anterior (and dorsal) neurons are called DN1a, they express high levels of CRY. More posterior, there are about 6-8 CRY-positive and 6-8 CRY-negative DN1p. Slightly ventral to the DN1 and just on top of the projections coming from the s-LNv are 2 neurons forming the DN2 group. In a more lateral and dorsal position there are about 40 neurons mainly of small size, the DN3 cluster. Finally there are 3 lateral posterior neurons (LPN) that are less characterised. DN2, DN3 (with a few exceptions) and LPN do not express CRY to visible levels (reviewed in Helfrich-Förster, 2003).

**Figure 6:**
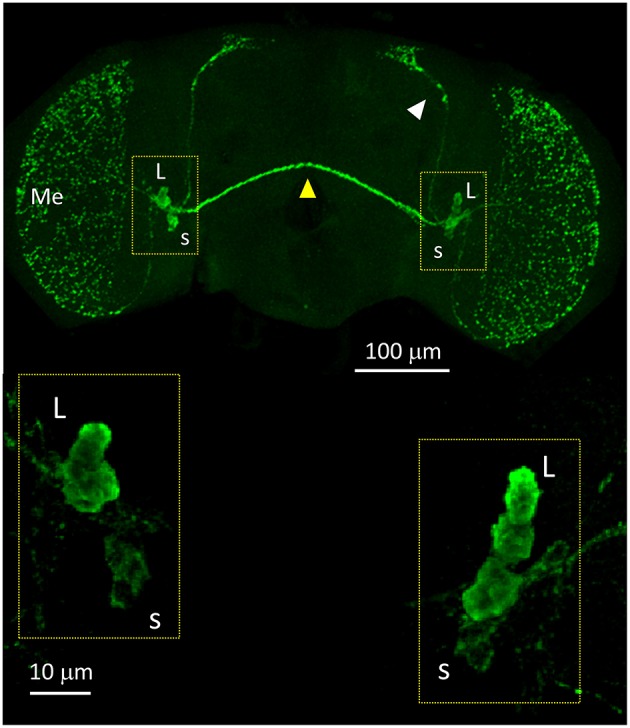
PDF immunoreactivity. Anti-PDF staining of a whole mount brain (ZT11). White arrow head = dorsal projections of the s-LNv (s). Yellow arrow head = posterior optic commissure connecting the l-LNv (L) of both sides. Additionally, the l-LNv project to the ipsilateral medulla (Me). The bottom panels show the LNv at higher magnification. All pictures are confocal maximum projections of several z-stacks. Contrast and brightness have been optimised for each panel as a whole.

We started the anatomical investigation of the circadian neuronal network in *D. suzukii* males by testing anti-PDF immunoreactivity (all antibodies used in this study had been raised against *D. melanogaster* proteins and previously validated). In *D. melanogaster*, anti-PDF staining is a convenient cytoplasmic marker of the LNv but it is also useful to infer the identity of the other neuronal clusters by their relative positions. We detected anti-PDF staining in four l-LNv and four s-LNv. The l-LNv projected to the ipsilateral and, *via* the posterior optic commissure, to the contralateral medulla. The s-LNv projected to the ipsilateral dorsal-posterior protocerubrum. The arrangement is very similar to the one described in *D. melanogaster*, although in *D. suzukii* the two LNv groups lie much closer together (Figure 6 and Figure S4).

**Figure 7:**
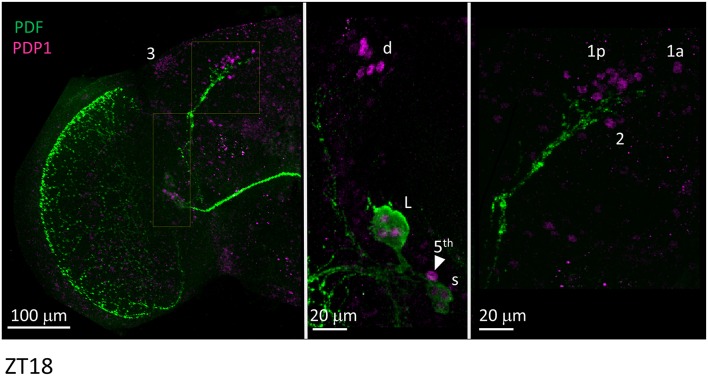
PDP1ε immunoreactivity. Flies were collected at ZT18. PDF immunoreactivity is in green, PDP1ε immunoreactivity is in magenta. The right and the middle panels are higher magnifications of the regions highlighted on the left panel. 3 = DN3. d = LNd. L = l-LNv. Arrow head and 5th = 5th-LN. s = s-LNv. 1p = DN1p. 1a = DN1a. 2 = DN2. Each panel represents confocal maximum projections of several z-stacks. Contrast and brightness have been optimised, modifications were applied to each panel as a whole.

We then applied anti-PDP1ε at ZT18 (*Zeitgeber* Time 18, with ZT0 = lights on and ZT12 = lights off), corresponding to the peak of PDP1ε expression in *D. melanogaster* (Cyran et al., 2003). We could recognise all putative clusters of clock neurons in *D. suzukii* with the exception of the LPN, which we were unable to identify unambiguously. Moreover, the numbers of neurons we observed per cluster were in agreement with those expected based on the *D. melanogaster* model. The only possible exception were the DN1a that we could not detect in half of the samples we analysed. Additionally, due to their small size and number we did not quantify the “crowded” DN3 cluster (Figure 7, Table 4).

**Table 4:** Identification of putative clock cells by immunolabelling.

**Protein**	**Average number of stained cells** **±** **Standard Deviation [Number of hemispheres]**								
	**ZT**	**s-LNv**	**l-LNv**	**5th-LNv**	**LNd**	**DN2**	**DN1a**	**DN1p**	**DN3**
PER	23	3.8 ± 0.4 [6]	4.0 ± 0.0 [6]	1.0 ± 0.0 [6]	5.4 ± 0.9 [5]	1.3 ± 0.6 [11]	1.9 ± 0.6 [11]	7.1 ± 0.8 [11]	^*^ [11]
PER	11	0.0 ± 0.0 [8]	0.0 ± 0.0 [8]	0.0 ± 0.0 [8]	0.0 ± 0.0 [8]	0.0 ± 0.0 [8]	0.0 ± 0.0 [8]	0.0 ± 0.0 [8]	0.0 ± 0.0 [8]
TIM	23	3.9 ± 0.4 [14]	4.1 ± 0.3 [14]	1.0 ± 0.0 [14]	5.7 ± 0.8 [13]	0.3 ± 0.5^a^ [6]	0.0 ± 0.0 [6]	6.8 ± 1.0 [6]	^*^ [6]
TIM	11	0.0 ± 0.0 [5]	0.0 ± 0.0^b^ [5]	0.0 ± 0.0^c^ [5]	0.0 ± 0.0^d^ [5]	0.0 ± 0.0 [6]	0.0 ± 0.0 [6]	0.0 ± 0.0 [6]	0.0 ± 0.0 [6]
PDP	18	3.9 ± 0.4 [8]	4.0 ± 0.0 [8]	0.9 ± 0.4 [8]	6.0 ± 0.0 [8]	1.9 ± 0.3 [12]	1.0 ± 1.0^e^ [12]	14.8 ± 1.0 [12]	^*^ [12]
CRY	23^∧^	3.8 ± 0.4 [5]	4.0 ± 0.0 [5]	1.0 ± 0.0 [5]	3.8 ± 0.4^f^ [5]	0.0 ± 0.0 [4]	0.0 ± 0.0 [4]	6.0 ± 0.0 [4]	0.0 ± 0.0 [4]
CRY	11	0.0 ± 0.0 [5]	0.0 ± 0.0 [5]	0.0 ± 0.0 [5]	0.0 ± 0.0 [5]	0.0 ± 0.0 [5]	0.0 ± 0.0 [5]	0.0 ± 0.0 [5]	0.0 ± 0.0 [5]

* *, staining observed but number of cells not quantified*.

∧ *, flies were kept 3 days in DD and then dissected at CT23*.

a *, 1 putative DN2 neuron was observed in two hemispheres only*.

b *, very weak and cytoplasmic staining was observed in 4 l-LNv in 2 hemispheres*.

c *, very weak and cytoplasmic staining was observed in 1 hemisphere*.

d *, very weak and cytoplasmic staining was observed in 2 LNd in 3 hemispheres*.

e *, 2 putative DN1a neurons were observed in 6 hemispheres only*.

f *, In two hemispheres 2 additional cells showing fainter staining were also observed*.

**Figure 8:**
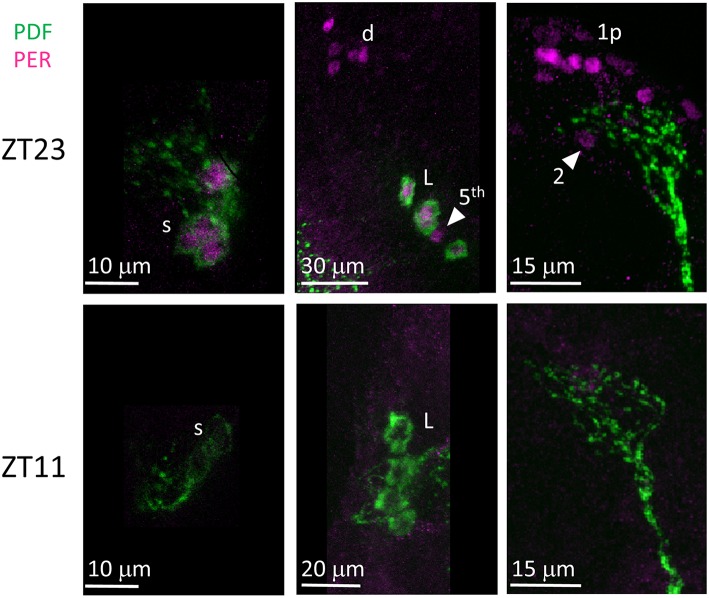
PER immunoreactivity. Flies were collected at ZT23 (top panels) and ZT11 (bottom panels). PDF immunoreactivity is in green, PER immunoreactivity is in magenta. PER immunoreactivity was detected at ZT23 but not at ZT11. s = s-LNv. d = LNd. L = l-LNv. Arrow head and 5th = 5th-LN. 1p = DN1p. 2 = DN2. Each panel represents confocal maximum projections of several z-stacks. Contrast and brightness have been optimised, modifications were applied to each panel as a whole.

**Figure 9:**
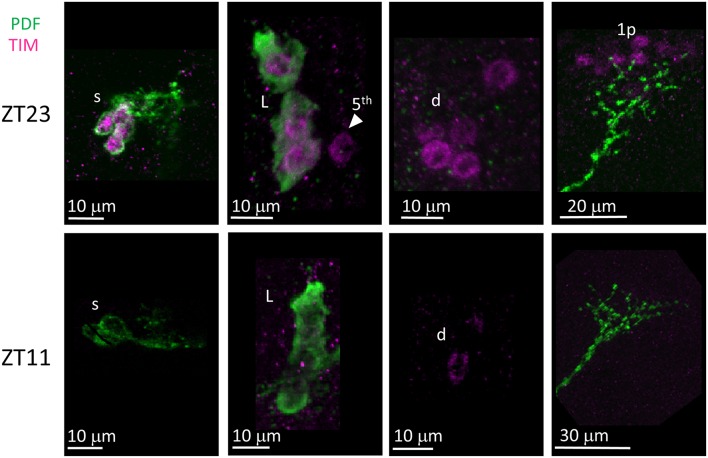
TIM immunoreactivity. Flies were collected at ZT23 (top panels) and ZT11 (bottom panels). PDF immunoreactivity is in green, TIM immunoreactivity is in magenta. TIM immunoreactivity was detected at ZT23 but not ZT11, a part from very weak cytoplasmic staining seldom seen in LN (for instance, second panel from the right on the bottom). s = s-LNv. L = l-LNv. Arrow head and 5th = 5th-LN. d = LNd. 1p = DN1p. Each panel represents confocal maximum projections of several z-stacks. Contrast and brightness have been optimised, modifications were applied to each panel as a whole.

Unlike many other *Drosophila* species (Hermann et al., 2013), we discovered that the clock neurons of *D. suzukii* can be labelled by anti-PER and anti-TIM antibodies raised against *D. melanogaster* proteins. We detected both immunoreactivities at ZT23 but not at ZT11 (Figures 8, 9). This is in agreement with the TTL model and increases our confidence on the specificity of the immunoreaction. We observed the expected numbers of LN; however the DN were more variable. We detected putative DN2 (often just 1 neuron) and DN1a with anti-PER but not with anti-TIM, and with both antibodies we observed just half the number of the putative DN1p revealed by anti-PDP1ε staining (Table 4).

**Figure 10:**
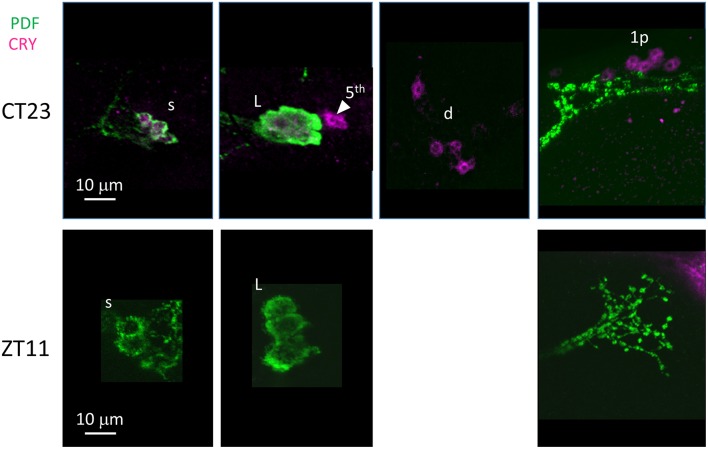
CRY immunoreactivity. Flies were collected at CT23 after 3 days in darkness (top panels) and ZT11 (bottom panels). PDF immunoreactivity is in green, CRY immunoreactivity is in magenta. CRY immunoreactivity was detected at CT23 but not ZT11.L = l-LNv. Arrow head and 5th = 5th-LN. d = LNd. s = s-LNv. 1p = DN1p. Each panel represents confocal maximum projections of several z-stacks. Contrast and brightness have been optimised, modifications were applied to each panel as a whole.

Finally we examined anti-CRY immunoreactivity (Figure 10). CRY is degraded by light in *D. melanogaster* (Stanewsky et al., 1998). Thus, to improve detection we kept *D. suzukii* flies in DD for 3 days before dissection at CT23 (Circadian Time 23, CT0 = subjective lights on, CT12 = subjective lights off). The LN showed the most prominent immunoreactivity; we observed staining in all LNv and in 4 LNd. In the dorsal brain, 6 DN1p showed CRY-immunoreactivity but we did not observe the expected staining in the DN1a (Table 4). As expected we did not observe CRY staining at ZT 11 (Figure 10, Table 4).

## Discussion

The first overall conclusion of this work is that *D. suzukii* is a challenging species for chronobiology studies in the laboratory. The flies do not survive well the standard laboratory conditions used for measuring single-fly locomotor activity, and they move little (Tables 1–3). This probably contributes to the large variability in the free run (i.e., DD and constant temperature) period seen after each entrainment condition, which becomes even more pronounced at lower temperatures for both males and virgin females (Figure 2C). At 18°C the difference in period between individuals at the extremes of the distribution can be a remarkable 10–15 h, suggesting that at least for this strain, in the laboratory and under cooler conditions, these flies appear to have very poor temperature compensation. This goes against what it is expected for a fundamental property of the clock with adaptive value (Pittendrigh, 1993; Sawyer et al., 1997). Such a result is even more surprising considering that these flies have evolved in a temperate habitat (Ometto et al., 2013) while the clock of *D. melanogaster*, of tropical origin (Mansourian et al., 2018), is well-adapted to 18°C. Our interpretation is that at 18°C the single-fly locomotor activity assay is not able to capture the true nature of the behaviour in *D. suzukii*, probably because the very low level of activity makes the determination of period imprecise. Entrainment is also different between the two species. Whereas*, D. melanogaster* are crepuscular, showing higher activity around the D/L (morning, M) and L/D (evening, E) switches, *D. suzukii* are mainly diurnal in our assay, being most active during the day (Figure 2D). In addition, the average day activity profiles do not show clear M and E peaks (with the exception of the hot conditions, LD 12:12, 28°C) and they are not consistently different between males and virgin females. In contrast, in *D. melanogaster* the day activity profile is a reliable predictor of gender (Figure 2D, Figure S2). We observed robust differences between virgin and mated females. Once mated, *D. suzukii* females show less variation in the circadian period of locomotor activity (Figure 3B) and a relative increase in day activity (Figures 3C,D). The latter had been observed before using group monitoring (4 flies tested together with LAM16 activity monitors, Trikinetics, USA) under both rectangular and seminatural conditions, which supports the robustness of this observation (Ferguson et al., 2015). We speculate that changes in both the entrainment profile (under LD) and the variance of the period (under DD) might underline a different coupling between clock neurons in females as a consequence of mating. Although we observed the same general trend in our *D. melanogaster* control (M1206) differences were not significant, possibly due to sample size or to genetic background (Figures 3A–D).

The low level of activity shown by *D. suzukii* under rectangular (on-off) light entrainment and single-fly monitoring motivated us to test alternative settings. We used custom-modified incubators producing so called seminatural conditions, such that light (of appropriate spectral composition) and temperature change gradually and in coordination simulating the natural environment (Green et al., 2015). We mimicked summer days in Northern Italy (where we collected our flies) using LD 16:8 and two temperature cycles, 20–30°C and 25–35°C. Under seminatural conditions *D. suzukii* males monitored individually still moved little and just during the day (Figures 4A,B). The absence of evening activity was particularly pronounced under the hotter cycle (25–35°C). Furthermore, the lack of plasticity in the regulation of their locomotor behaviour was even more severe than under rectangular light entrainment (Figure 2D) and also compared to published observations where single-fly rhythmicity was tested under conditions mimicking summer (LD 14:10, 12.2–22.2°C) and winter (LD 11:13, 6.8–16.7°C) in Watsonville California, the site of the first *D. suzukii* detection in North America (Hamby et al., 2013). In addition, our results disagree with data from the wild (unmanaged blueberry fields in Bacon County, Georgia, US) obtained by trapping, suggesting that *D. suzukii* is mainly a crepuscular species that modulates activity in response to temperature and humidity fluctuations (Evans et al., 2017).

The *D. melanogaster* males we tested under seminatural conditions manifested the opposite behaviour, robust and persistent locomotion, further increased under the most challenging condition (Figures 4A,B). Under 20–30°C cycles *D. melanogaster* males were able to pace their locomotion according to the interactions between light and temperature, avoiding being active during the hottest part of the day (Figure 4A). However, such a process partially failed under 25–35°C cycles, giving way to a large burst of day activity called the afternoon peak, which is likely a sustained escape response (Figure 4B) (Vanin et al., 2012; Green et al., 2015). Extrapolating from these results we hypothesised that the two species might adopt a different strategy to overcome stressful environmental conditions, with *D. suzukii* suppressing activity and *D. melanogaster* increasing it. This is relevant because reduced locomotion can give the impression of a weak clock when in reality a low activity output might not reflect fairly its circadian regulation. To investigate this further we performed preliminary experiments where 10 males or 10 males and 10 females were tested together under LD 12:12, 25°C in a growth vial using a population monitor (DPM, Tikinetics, USA) (Figures 5A,B). Conceptually similar experiments but using different settings and equipment had been performed before in both *D. melanogaster* and *D. suzukii* (Levine et al., 2002; Fujii et al., 2007; Ferguson et al., 2015; Shaw et al., 2018a). One general conclusion that applies to the published work and to our own is that the presence of conspecifics influences the activity profile and thus social interactions are an important *Zeitgeber* for the circadian clock. Moreover, housing *D. melanogaster* males and females in close proximity results in increased nocturnal behaviour (Fujii et al., 2007). We have confirmed those conclusions and extended them to *D. suzukii* (Figure 5B). However, our most interesting observation is that when tested in a socially rich environment, *D. suzukii* flies seem much more active and synchronous than when tested in isolation (Figures 5A,B; Figures S2, S3). A caveat is that our results are still preliminary and limited to LD entrainment. However, they agree with an interesting study where 10 males and 10 females were monitored together on nitrogen-free medium for about 6 days in LAM25 monitors (Trikinetics, USA). The authors recreated in the laboratory temperature and light conditions found in East Malling (Kent, UK) during June (LD 18:6, 11–22°C), August (LD 16:8, 14–32°C) and October (LD 12.5:11.5, 9–18°C) 2016. The flies displayed a good level of locomotion but also they paced activity as a function of the interaction between light and temperature (Shaw et al., 2018a; Figure 9 therein). We never observed such a plastic behaviour in *D. suzukii* flies monitored individually. Conversely, *D. melanogaster* flies were able to retain plasticity even in isolation. Therefore, our results are a warning that for some species isolation might not be permissive to the manifestation of locomotor activity rhythms as a robust and reliable experimental window to the clock, something that we take for granted. We anticipate that by testing *Drosophila* species under seminatural-socially enriched conditions we will improve the representation of the natural environment in the laboratory and gain a better understanding of the adaptive value of the clock.

Finally, we asked whether the behavioural differences we observed between the two species would correlate with differences in the organisation of the neuronal network of the clock. To identify putative clock neurons in *D. suzukii* we used well-characterised antibodies made against *D. melanogaster* clock proteins that we had tested before (see Materials and Methods for antibodies information). In *D. melanogaster* the clock neurons can be subdivided into lateral and dorsal neurons. The former consist of three clusters (s-LNv, l-LNv, and LNd) and a single neuron (5th-LNv). The LN are easily recognizable: they are quite predictable in anatomical location, in size and in relative position to each other and to anatomical landmarks. Moreover, the number of neurons in each cluster is low (1–6), they are not “tightly packed” and PDF—a clock relevant neuropeptide – is a specific marker for the two ventral groups (s-LNv and l-LNv) labelling their cytoplasm including projections. The identification of the DN is more ambiguous as they are more irregular in size and distribution, often they are very close to one another, they are numerous and we do not have a specific marker for them. We tested *D. suzukii* males for immunoreactivity against the neuropeptide PDF and the clock proteins PDP1ε, PER, TIM and CRY (Figures 6–10). The overall picture is that the neuronal organisation of the circadian network is quite similar in *D. suzukii* and *D. melanogaster*, but there are some differences. PDF is expressed in the LNv and we can see projections to the medulla and to the dorsal protocerebrum, reproducing the well-described pattern known for *D. melanogaster* (Figure 6). One difference is that in *D. suzukii* the l-LNv are much more tightly packed together and to the s-LNvs (Figure 6 and Figure S4; see also Figure 7, middle panel). Anti-PDP1ε staining at ZT18 (the time when PDP1ε–always nuclear—is at its maximum in *D. melanogaster*; Cyran et al., 2003) revealed that all major types of clock neurons are putatively present and in numbers comparable to *D. melanogaster*, with the proviso that we did not quantify the small and numerous DN3 neurons (more than 40 in *D. melanogaster*) and we could not unequivocally identify the LPN (Figure 7 and Table 4). Anti-PER and anti-TIM staining at ZT23 and ZT11 (respectively, when they are nuclear and highly abundant or absent in *D. melanogaster*) were also consistent with an overall conservation of the clock between *D. melanogaster* and *D. suzukii* (Figures 8, 9). The LN were clearly identified and the expression of the two proteins was nuclear and high or absent at the appropriate times. However, some differences in expression emerged in the DN. At ZT23 we usually observed only one (out of two) DN2 with anti-PER and none with anti-TIM staining. We were able to distinguish the two DN1a with anti-PER but not with anti-TIM. Lastly, only half the number of PDP1ε-positive DN1p showed anti-PER or anti-TIM staining (*ca*. 7 out of *ca*. 15). Double-labelling experiments will be necessary to distinguish whether the same or different DN1p show anti-PER and anti-TIM immunoreactivity and to prove co-localization with PDP1ε. The latter is particularly important as in *D. melanogaster* PDP1ε expression in not limited to clock cells and the same might occur in *D. suzukii* (Cyran et al., 2003). Curiously, at ZT11 we observed very weak anti-TIM (but not anti-PER) cytoplasmic staining in a few LN (Figure 9, bottom, second panel from the right and Table 4). In *D. melanogaster* PER and TIM start accumulating in the cytoplasm in the evening, they transition into the nucleus in the middle of the night and become fully nuclear by the end of dark phase. Then they disappear, with TIM being degraded faster than PER as TIM is directly targeted for degradation after exposure to light whereas the decline of PER is a consequence of it requiring TIM for stability (reviewed in Özkaya and Rosato, 2012). If this regulation were the same in *D. suzukii* it is unclear how the rise of TIM could lead that of PER. Time course experiments assessing the progressive accumulation and degradation of both proteins will be required to clarify this. Finally, we examined immunoreactivity to CRY (Figure 10 and Table 4). Assuming the protein might be subject to light-dependent degradation as in *D. melanogaster* we maintained the flies in darkness for three days prior to their collection at CT23. The immunosignal was consistent with what was expected in comparison with the *D. melanogaster* model with the exception that we did not observe staining in DN1a cells. Again, double-labelling experiments should be carried out to test whether or not the putative 6 CRY-positive DN1p additionally express PER and/or TIM. As expected we did not observe anti-CRY immunoreactivity at ZT11.

At the beginning of our discussion we highlighted the difficulties encountered when investigating the clock of this species. However, at the end of it our overall judgement is that *D. suzukii*, although challenging, is a model worthy of future chronobiology investigations. Glimpses suggest that some elements of the circadian neuronal network might be different from *D. melanogaster* and some molecular details might have evolved. More work is required before a clear picture can be drawn, but a better understanding of the clock of *D. suzukii* is in reach using the modern tools of genetic manipulation. The most interesting aspect will be investigating how social interactions impact on the clock and on the mechanisms that regulate arousal and stress responses in this fascinating species. We speculate that the evolution of lower arousal and of stress responses that promote inactivity have been instrumental in the ecological transitioning of the species. Although *D. suzukii* can successfully lay eggs in rotting fruits, they are outcompeted by other drosophilids (Shaw et al., 2018b). Being less aroused and less active under environmental stress might allow *D. suzukii* to save eggs and energy waiting for the next short burst of fruit ripening, when fresh fruits become available to lay their eggs without competition. Perhaps, their propensity for living at a “lower gear” is the secret of their current success as an invasive pest species.

## Data Availability

The raw data supporting the conclusions of this manuscript will be made available by the authors upon reasonable request.

## Author Contributions

All authors contributed to the generation of hypotheses and the interpretation of results. LG contributed materials. ER and CK contributed funding. CH, ÖÖ, and HR provided essential technical knowledge and solutions. CH and ER performed experiments and analysed data. CH and ER wrote the manuscript with input from all the authors.

### Conflict of Interest Statement

The authors declare that the research was conducted in the absence of any commercial or financial relationships that could be construed as a potential conflict of interest.

## Supplementary Material

Figure S1 Average day profile for *D. suzukii* males S1210, SMichele, S1203, and S1209 under standard laboratory conditions. The average day was obtained by averaging into one the four LD days for all rhythmic flies (SR+CR, see Table 1) of the same genotype. Black columns correspond to dark and white columns to light. Error bars show the standard deviation. Note that the error bars reaching the top of the graph are truncated. This reflects a compromise in the choice of the scale of the Y-axis to allow the appreciation of both the average activity profile and its variance. Activity levels (0–40 crossing/30 min) are shown on the Y-axis, time (48 intervals of 30 min) is on the X-axis. Note that the profile for S1210 is the same as in Figure 1B but at different scale on the Y-axis.

Figure S2 Combined LD activity under all entrainment conditions in *D. melanogaster* (M1217) and *D. suzukii* (S1202) virgin females (fv) and males (m). To test whether virgin females and males from the two species differ in their average activity under LD we combined 31 flies (the maximum common number of rhythmic flies across conditions, genders and species) at random for each entrainment condition. In M1217, virgin females (mean LD activity = 18.21 crossing/30 min) were more active than males (mean LD activity = 16.88 crossing/30 min); the difference is statistically supported (Kolmogorov-Smirnov test, ^***^*P* = 0.0003). In S1202, virgin females (mean LD activity = 3.55 crossing/30 min) and males (mean LD activity = 3.61 crossing/30 min) showed equal activity. Note that *D. melanogaster* flies are about five times more active than *D. suzukii*.

Figure S3 Population rhythms. Activity levels in 10 males *D. suzukii* (S1202) and 10 males *D. melanogaster* (M1217). Activity levels (number of crossing/30 min) have been plotted separately for the three detector rings (R1, R2, and R3) to give an impression of the overall activity. See also Table S1.

Figure S4 Anti-PDF staining of the LNv in *D. suzukii* and *D. melanogaster*. Typically, in *D. suzukii* the l-LNv are closer to one another and to the s-LNv than in *D. melanogaster*. L = l-LNv. s = s-LNv. Each panel represents confocal maximum projections of several z-stacks. Contrast and brightness have been optimised, modifications were applied to each panel as a whole.

Table S1 Descriptive statistics for amount of activity/bin. The locomotor activities of 10 D. suzukii males and 10 D. melanogaster males were recorded for 5 days under LD 12:12, 25°C.

Video 1 A simple startling stimulus, banging a growth vial on the bench, is applied to *D. suzukii* and *D. melanogaster* cultures. The locomotor response of the two species is different showing high arousal and activity in *D. melanogaster* and reduced arousal and activity in *D. suzukii*.
