# Supplementary figures and images for: Locomotor Behaviour and Clock Neurons Organisation in the Agricultural Pest Drosophila suzukii
Source: Front Physiol. 2019 Jul 24;10:941. doi: 10.3389/fphys.2019.00941 (PMC6667661; doi:10.3389/fphys.2019.00941)

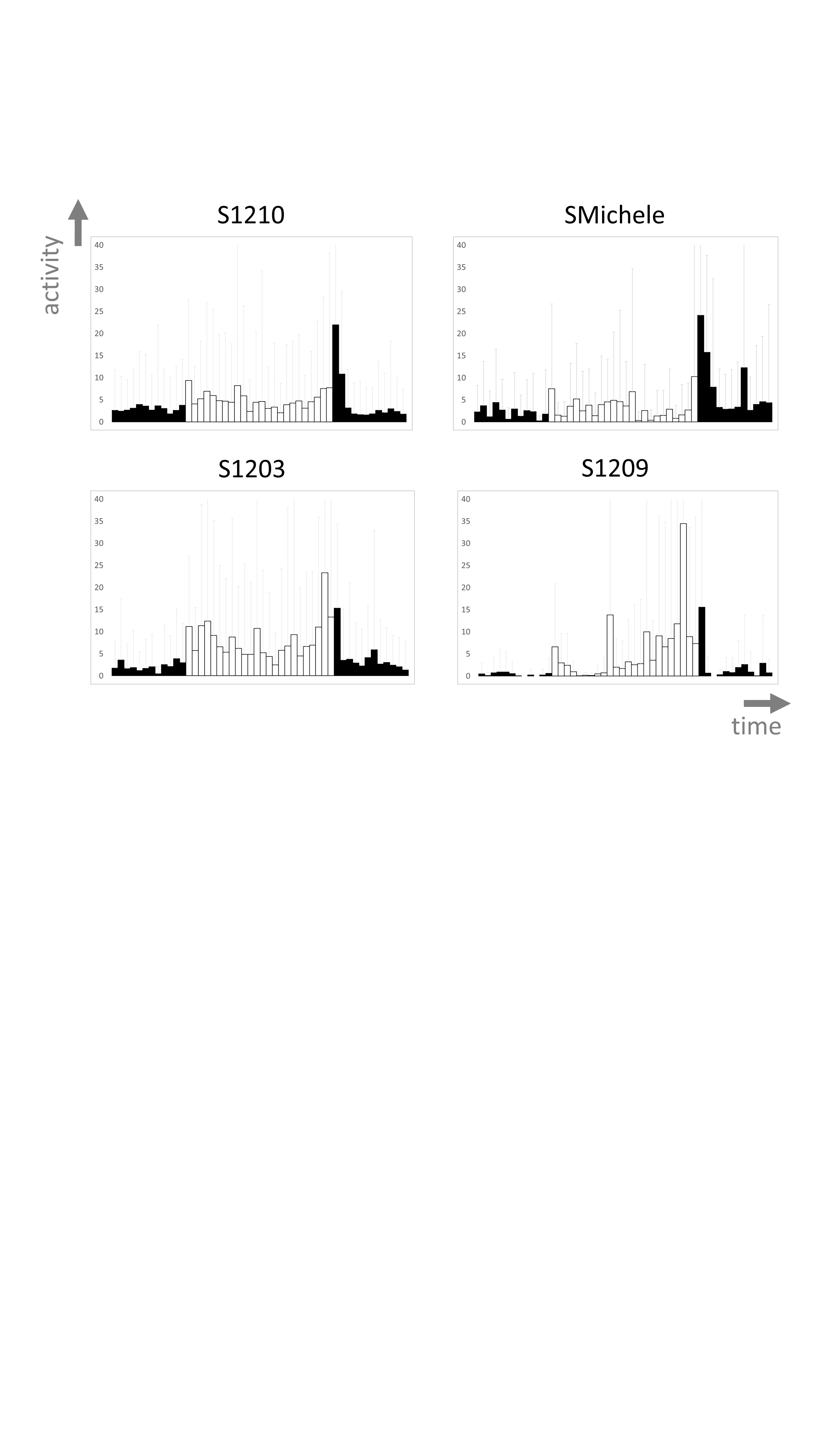

Supplement: Figure S1 — Average day profile for D. suzukii males S1210, SMichele, S1203, and S1209 under standard laboratory conditions. The average day was obtained by averaging into one the four LD days for all rhythmic flies (SR+CR, see Table 1) of the same genotype. Black columns correspond to dark and white columns to light. Error bars show the standard deviation. Note that the error bars reaching the top of the graph are truncated. This reflects a compromise in the choice of the scale of the Y-axis to allow the appreciation of both the average activity profile and its variance. Activity levels (0–40 crossing/30 min) are shown on the Y-axis, time (48 intervals of 30 min) is on the X-axis. Note that the profile for S1210 is the same as in Figure 1B but at different scale on the Y-axis. [file Image_1.TIF]

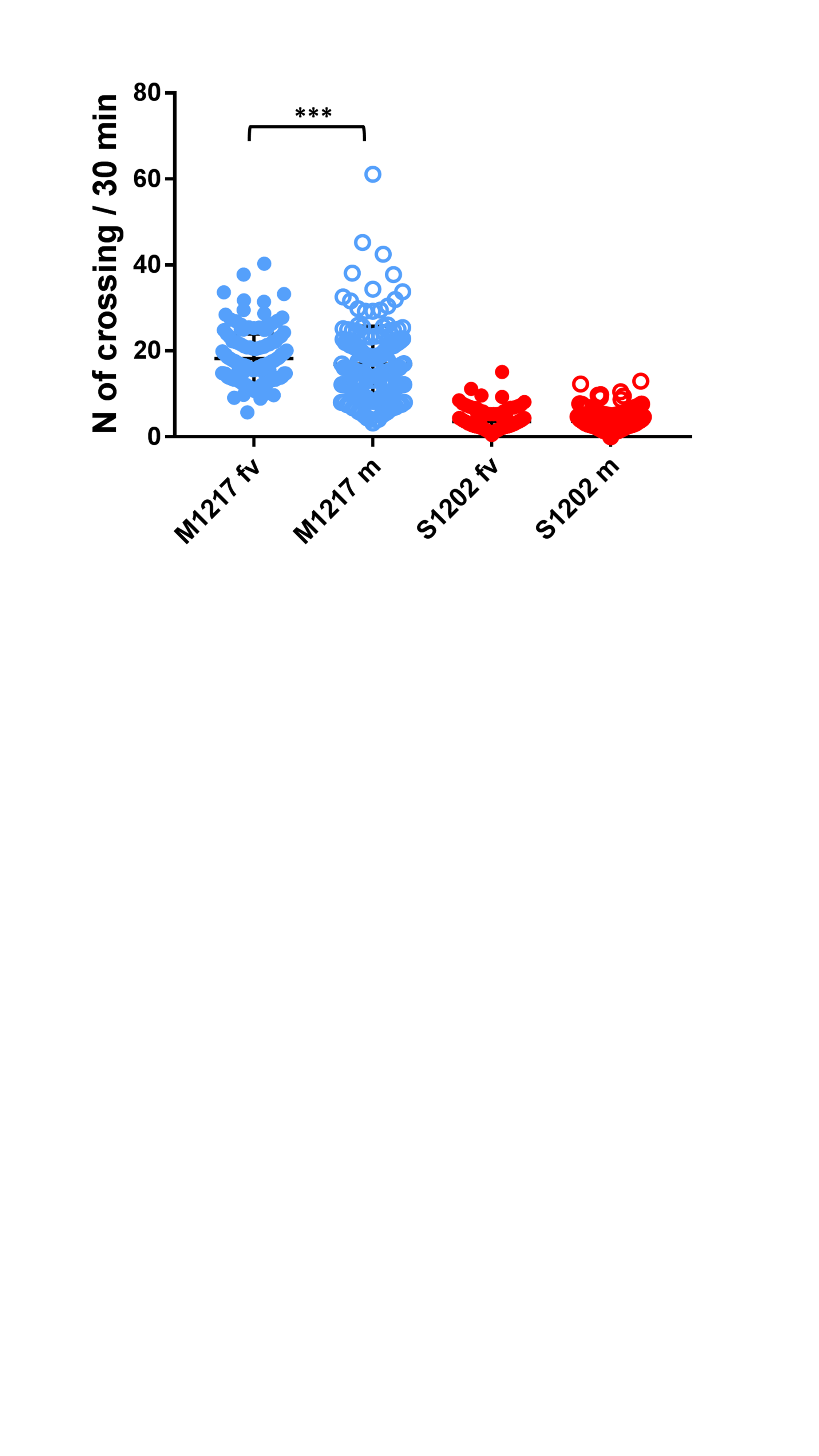

Supplement: Figure S2 — Combined LD activity under all entrainment conditions in D. melanogaster (M1217) and D. suzukii (S1202) virgin females (fv) and males (m). To test whether virgin females and males from the two species differ in their average activity under LD we combined 31 flies (the maximum common number of rhythmic flies across conditions, genders and species) at random for each entrainment condition. In M1217, virgin females (mean LD activity = 18.21 crossing/30 min) were more active than males (mean LD activity = 16.88 crossing/30 min); the difference is statistically supported (Kolmogorov-Smirnov test, ***P = 0.0003). In S1202, virgin females (mean LD activity = 3.55 crossing/30 min) and males (mean LD activity = 3.61 crossing/30 min) showed equal activity. Note that D. melanogaster flies are about five times more active than D. suzukii. [file Image_2.TIF]

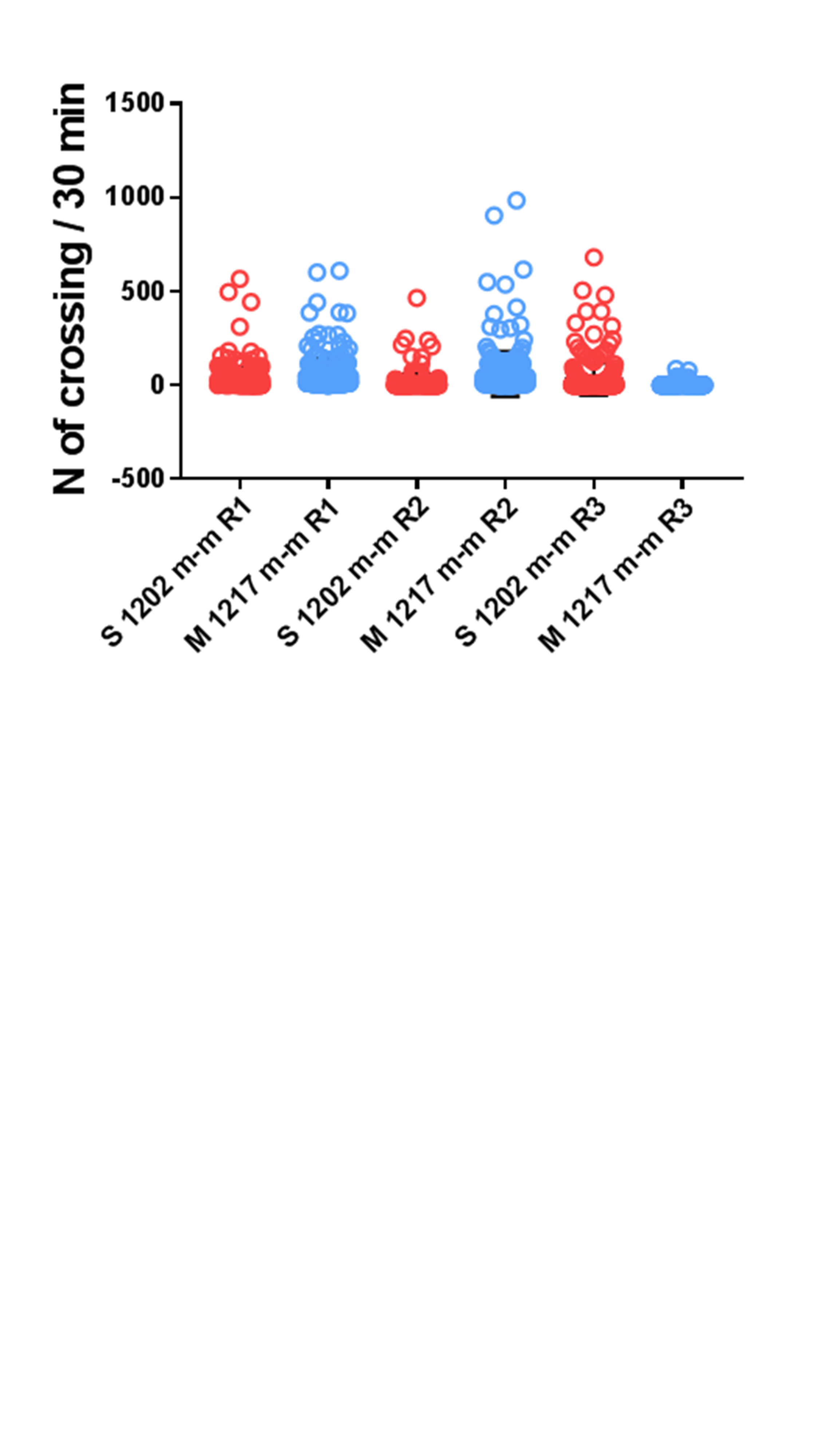

Supplement: Figure S3 — Population rhythms. Activity levels in 10 males D. suzukii (S1202) and 10 males D. melanogaster (M1217). Activity levels (number of crossing/30 min) have been plotted separately for the three detector rings (R1, R2, and R3) to give an impression of the overall activity. See also Table S1. [file Image_3.TIF]

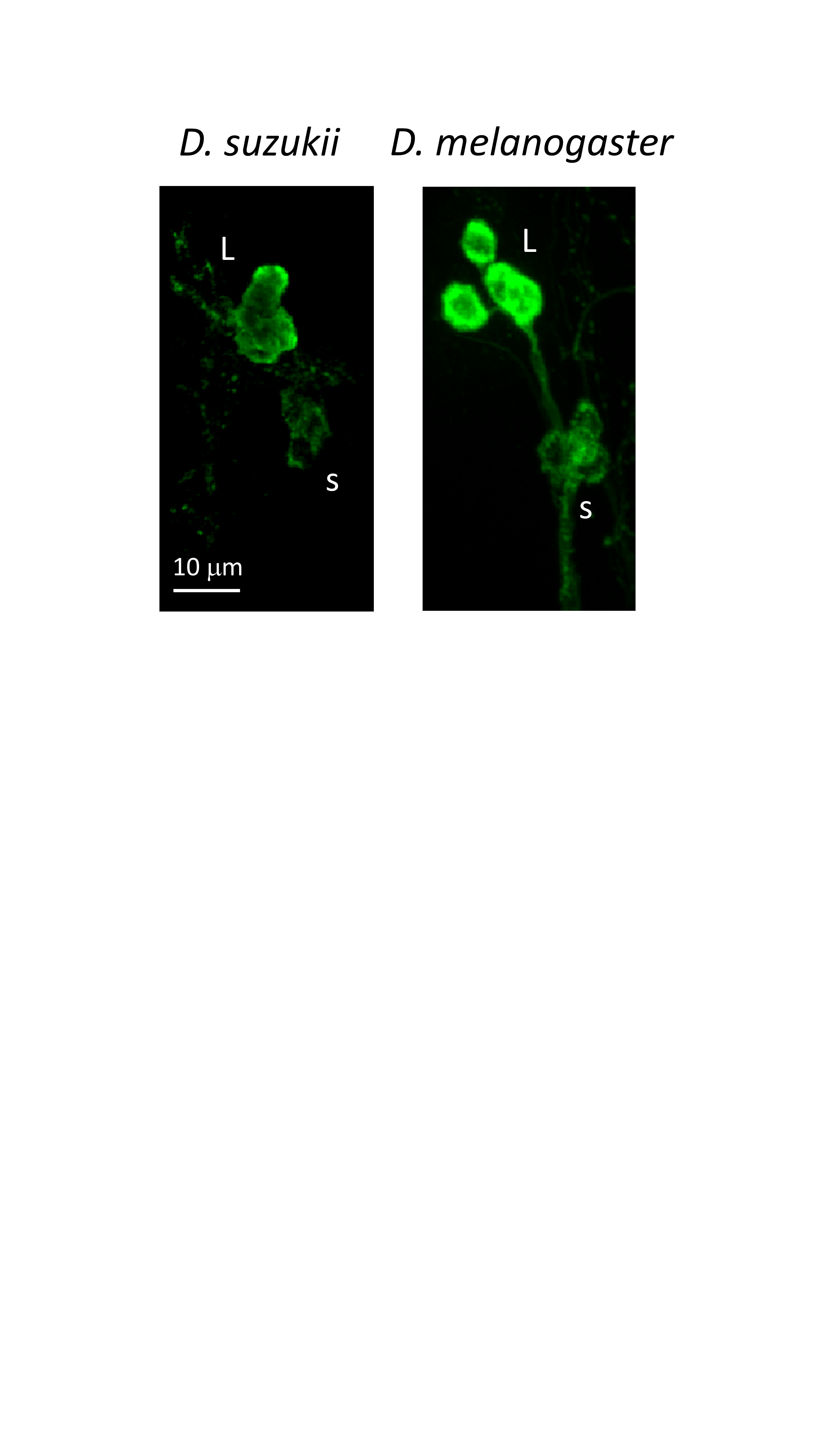

Supplement: Figure S4 — Anti-PDF staining of the LNv in D. suzukii and D. melanogaster. Typically, in D. suzukii the l-LNv are closer to one another and to the s-LNv than in D. melanogaster. L = l-LNv. s = s-LNv. Each panel represents confocal maximum projections of several z-stacks. Contrast and brightness have been optimised, modifications were applied to each panel as a whole. [file Image_4.TIF]
