# Supplementary material for: Locomotor Behaviour and Clock Neurons Organisation in the Agricultural Pest Drosophila suzukii
Source: Front Physiol. 2019 Jul 24;10:941. doi: 10.3389/fphys.2019.00941 (PMC6667661; doi:10.3389/fphys.2019.00941)
Supplement: Table S1 — Descriptive statistics for amount of activity/bin. The locomotor activities of 10 D. suzukii males and 10 D. melanogaster males were recorded for 5 days under LD 12:12, 25°C. [file Table_1.DOCX]

**TableS1. Descriptive statistics for amount of activity/bin. The locomotor activities of 10 *D. suzukii* males and10 *D. melanogaster* males were recorded for 5 days under LD 12:12, 25 °C.**

| **N crossing/30min** | **S 1202 m-m** | | | **M 1217 m-m** | | |
| --- | --- | --- | --- | --- | --- | --- |
|  |  |  |  |  |  |  |
|  | **R1** | **R2** | **R3** | **R1** | **R2** | **R3** |
| **Number of Bins** | 241 | 241 | 241 | 241 | 241 | 241 |
|  |  |  |  |  |  |  |
| **Minimum** | 0 | 0 | 0 | 0 | 0 | 0 |
| **25% Percentile** | 0.33 | 0 | 0 | 10 | 7 | 0 |
| **Median** | 8.67 | 1 | 1.33 | 24 | 24 | 0 |
| **75% Percentile** | 38.83 | 7.835 | 15.5 | 59 | 56 | 0 |
| **Maximum** | 568.3 | 466.3 | 683 | 611 | 986 | 87 |
|  |  |  |  |  |  |  |
| **Mean** | 33.14 | 14 | 32.31 | 55.24 | 60.32 | 1.83 |
| **Std. Deviation** | 67.58 | 45.47 | 87.3 | 86.63 | 119.3 | 9.088 |
| **Std. Error of Mean** | 4.353 | 2.929 | 5.624 | 5.581 | 7.684 | 0.5854 |
|  |  |  |  |  |  |  |
| **Lower 95% CI of mean** | 24.56 | 8.228 | 21.24 | 44.25 | 45.18 | 0.6767 |
| **Upper 95% CI of mean** | 41.71 | 19.77 | 43.39 | 66.24 | 75.45 | 2.983 |
|  |  |  |  |  |  |  |
| **Sum** | 7987 | 3374 | 7788 | 13314 | 14536 | 441 |
